# c-di-GMP Homeostasis Is Critical for Heterocyst Development in *Anabaena* sp. PCC 7120

**DOI:** 10.3389/fmicb.2021.793336

**Published:** 2021-12-03

**Authors:** Min Huang, Ju-Yuan Zhang, Xiaoli Zeng, Cheng-Cai Zhang

**Affiliations:** ^1^State Key Laboratory of Freshwater Ecology and Biotechnology, Key Laboratory of Algal Biology, Institute of Hydrobiology, Chinese Academy of Sciences, Wuhan, China; ^2^College of Advanced Agricultural Sciences, University of Chinese Academy of Sciences, Beijing, China; ^3^Institut AMU-WUT, Aix-Marseille University and Wuhan University of Technology, Wuhan, China; ^4^Innovation Academy for Seed Design, Chinese Academy of Sciences, Beijing, China

**Keywords:** cyanobacteria, c-di-GMP, nitrogen fixation, signal transduction, homeostasis

## Abstract

c-di-GMP is a ubiquitous bacterial signal regulating various physiological process. *Anabaena* PCC 7120 (*Anabaena*) is a filamentous cyanobacterium able to form regularly-spaced heterocysts for nitrogen fixation, in response to combined-nitrogen deprivation in 24h. *Anabaena* possesses 16 genes encoding proteins for c-di-GMP metabolism, and their functions are poorly characterized, except *all2874* (*cdgS*) whose deletion causes a decrease in heterocyst frequency 48h after nitrogen starvation. We demonstrated here that c-di-GMP levels increased significantly in *Anabaena* after combined-nitrogen starvation. By inactivating each of the 16 genes, we found that the deletion of *all117*5 (*cdgSH*) led to an increase of heterocyst frequency 24h after nitrogen stepdown. A double mutant *ΔcdgSHΔcdgS* had an additive effect over the single mutants in regulating heterocyst frequency, indicating that the two genes acted at different time points for heterocyst spacing. Biochemical and genetic data further showed that the functions of CdgSH and CdgS in the setup or maintenance of heterocyst frequency depended on their opposing effects on the intracellular levels of c-di-GMP. Finally, we demonstrated that heterocyst differentiation was completely inhibited when c-di-GMP levels became too high or too low. Together, these results indicate that the homeostasis of c-di-GMP level is important for heterocyst differentiation in *Anabaena*.

## Introduction

Cyclic nucleotides act as second messengers in all domains of life (Tschowri et al., 2014). One of them, bis-(3'-5')-cyclic dimeric GMP (c-di-GMP), has emerged as one of the most common and important secondary messengers in bacteria (Romling et al., 2013). It was first discovered in *Gluconacetobacter xylinus* about 30years ago, as a positive allosteric regulator of cellulose synthase (Ross et al., 1987). Subsequent studies have revealed that c-di-GMP regulates a variety of important cellular processes, including biofilm formation, motility, virulence, cell differentiation, cell cycle and mechanical sensing (Tischler and Camilli, 2004; Kulesekara et al., 2006; Hengge, 2009; Boehm et al., 2010; Romling et al., 2013; Tschowri et al., 2014; Lori et al., 2015; Hug et al., 2017; Xu et al., 2019; Del Medico et al., 2020; Snyder et al., 2020). Such regulations may take place at the transcriptional, post-transcriptional, or post-translational levels by interacting with different types of effectors (Romling et al., 2013; Chou and Galperin, 2016). Several classes of c-di-GMP receptors, such as PilZ domain-containing receptors, I site receptors, inactive EAL domain receptors and riboswitches, can be identified by sequence alignment, since they usually share a conserved c-di-GMP binding motif (Romling et al., 2013; Chou and Galperin, 2016). However, other newly discovered receptors are being identified, which have no common features in protein sequences (Hickman and Harwood, 2008; Burdette et al., 2011; Tschowri et al., 2014; Lori et al., 2015; Xu et al., 2019; Gallagher et al., 2020).

The intracellular level of c-di-GMP is attributed to two opposing enzymatic activities: diguanylate cyclase (DGC) and c-di-GMP-specific phosphodiesterase (PDE). DGCs contain the conserved GG(D/E)EF motif and can synthesize one molecule of c-di-GMP using two GTPs as substrate (Chan et al., 2004; Paul et al., 2007). PDEs harbor either the EAL or the HD-GYP domain and can hydrolyze c-di-GMP into 5'-phosphoguanylyl-(3'-5')-guanosine (pGpG) or further down to GMP (Schmidt et al., 2005; Christen et al., 2006). GG(D/E)EF, EAL or HD-GYP domains may co-occur in multi-domain proteins, together with other diverse regulatory domains, including REC (Receiver), PAS (Per–ARNT–Sim), GAF (cGMP phosphodiesterase/adenylate cyclase/FhlA), FHA (fork head-associated), and CHASE (cyclase/histidine kinases-associated sensing extracellular). These regulatory domains control the synthesis or degradation of c-di-GMP in response to specific environmental cues, such as oxygen, light, quorum sensing signals, aromatic hydrocarbons, phosphorylation and proton flux (Paul et al., 2004; Barends et al., 2009; Tuckerman et al., 2009; Savakis et al., 2012; Martin-Moldes et al., 2016; Hug et al., 2017; Yang et al., 2017). Bioinformatics analysis of completed cyanobacterial genomes indicate that most of them contain multiple genes related to c-di-GMP turnover (Romling et al., 2013). This observation suggests that, while c-di-GMP has important functions in cyanobacteria, its exact roles remain poorly characterized. In the unicellular cyanobacterium *Synechocystis* sp. PCC 6803, the intracellular level of c-di-GMP regulates phototaxis (Savakis et al., 2012). In *Thermosynechococcus vulcanus*, the intracellular c-di-GMP level is controlled by a light-quality sensitive input system formed by three cyanobacteriochromes and regulates formation of cell aggregation (Enomoto et al., 2014, 2015).

When grown under diazotrophic conditions, the filamentous cyanobacterium *Anabaena*/*Nostoc* PCC 7120 (*Anabaena*) has two types of cells, vegetative cells and heterocysts. Vegetative cells can divide and perform photosynthesis, whereas heterocysts are terminally differentiated, nitrogen-fixing cells (Wolk et al., 1994; Kumar et al., 2010; Muro-Pastor and Hess, 2012; Herrero and Flores, 2019). Heterocysts represent 5–10% of all cells along the filaments (Wolk et al., 1994; Kumar et al., 2010; Muro-Pastor and Hess, 2012; Herrero and Flores, 2019), and are formed in response to deprivation of combined nitrogen. Single heterocysts are semi-regularly spaced along the filaments, and the existence of the two cell types allows *Anabaena* filaments to perform simultaneously two incompatible activities, oxygen-labile nitrogen fixation and oxygen-evolving photosynthesis. Heterocysts supply vegetative cells with fixed nitrogen, and in return receive fixed carbon from vegetative cells (Wolk et al., 1994; Kumar et al., 2010; Muro-Pastor and Hess, 2012; Herrero and Flores, 2019). Heterocyst development and pattern formation are complex and highly coordinated processes, during which several signals and a large number of positive or negative regulators are involved (Zhang et al., 2006). The signals include peptides derived from PatS, PatX, HetN, serving as inhibitors for formation and maintenance of heterocyst pattern (Yoon and Golden, 1998; Callahan and Buikema, 2001; Elhai and Khudyakov, 2018), 2-oxoglutarate whose accumulation acts as a nitrogen starvation signal and a trigger for heterocyst development (Laurent et al., 2005), and ppGpp required for heterocyst differentiation (Zhang et al., 2013).

*Anabaena* possesses 16 genes encoding proteins with GG(D/E)EF motif or EAL/HD-GYP motif; however, the physiological functions of c-di-GMP have not been well explored. A report by Neunuebel and Golden showed that the inactivation of *all2874* (*cdgS:* c-di-GMP synthetase) encoding a diguanylate cyclase caused a significant reduction in heterocyst frequency at the maintenance steps (Neunuebel and Golden, 2008). In this mutant, the heterocyst frequency was similar to the wild type at the initial formation step, but over the next 4days, it was decreased dramatically as compared with wild type. The intervals between heterocysts increased to as many as 200 vegetative cells, compared to less than 25 vegetative cells for the control (Neunuebel and Golden, 2008). The relationship between intracellular c-di-GMP levels and heterocysts development, and the underlying mechanism, are still unclear.

Here, we report that c-di-GMP plays a critical role in regulating heterocyst development. Our data show that c-di-GMP levels in *Anabaena* accumulate transiently after nitrogen starvation. By combining genetic and biochemical analyses, we established the relationship between c-di-GMP levels and heterocysts frequency under the control of two genes, *all117*5 (*cdgSH*: c-di-GMP synthetase and hydrolase) and *all2874* (*cdgS*). We further demonstrated that the homeostasis of c-di-GMP is important for heterocyst differentiation in *Anabaena*. This study paves the way for characterizing the signalling function of c-di-GMP in this cyanobacterium, a model for prokaryotic development.

## Materials and Methods

### Strains and Culture Conditions

All strains used in this study are listed in Supplementary Table S1. *Anabaena* WT and its derivatives were grown in BG11 (Stanier et al., 1971). For heterocyst induction, cells grown to logarithmic phase in BG11 were transferred to BG11_0_ (BG11 without combined nitrogen) medium as described previously (Golden et al., 1991). All strains were cultured in liquid medium at 30°C with shaking at 180rpm and the constant illumination with light intensity at 30μmolm^−2^ s^−1^, the CO_2_ concentration is 400ppm. The overexpression strains based on the artificial CT promoter (Xing et al., 2020) were grown first in BG11 medium to logarithmic phase, then transferred to BG11_0_ in the presence of copper and theophylline, for the induction of heterocysts development and gene expression. When not specified, the concentration of the copper and theophylline is 0.5 and 2mM, respectively. To modify the c-di-GMP level *in vivo*, gradient concentrations of copper/theophylline were tested at: 0μM/0mM, 0.125μM/0.5mM, 0.25μM/1mM, 0.5μM/2mM, and 1μM/4mM. Whenever necessary, neomycin (25μg/ml), streptomycin (2.5μg/ml) or spectinomycin (5μg/ml) was supplied in BG11 or BG11_0_ medium.

### Construction of Plasmids and Cyanobacterial Recombinant Strain

All plasmids used in this study are listed in Supplementary Table S2 and verified by Sanger sequencing. All the oligonucleotides used for plasmid construction and mutant checking in this study are listed in Supplementary Table S3.

All mutant stains with markless deletion of the corresponding genes were generated in this study by the genome editing technique based on CRISPR-Cpf1, and the plasmids were constructed as previously described (Niu et al., 2019). The plasmids for gene overexpression in *Anabaena* were constructed based on the PCT vector (Xing et al., 2020), allowing control of gene expression by the artificial CT promoter. This promoter was consisted of the *petE* promoter region and a theophylline riboswitch. Gene expression could thus be induced by adding copper and theophylline in the culture media. The plasmids used for protein purification from *E. coli* BL21 (DE3) were constructed based on the pHTwinStrep vector, which containing two carboxyl-terminals Strep-tag at the C-terminal.

To construct the mutant and overexpression strains, the corresponding plasmid was transferred into *Anabaena* by conjugation through triparental mating as previously described (ElhaI et al., 1997). All mutants and overexpression strains were confirmed by PCR.

### Extraction and Quantification of Cellular c-di-GMP

c-di-GMP in *Anabaena* was analyzed as previously described (Agostoni et al., 2013). At the indicated time points or the indicated conditions, 2ml of cells were quickly collected by filtering under standard light illumination and resuspended with 500μl ice-cold extraction buffer containing 40% acetonitrile, 40% methanol, and 0.1N formic acid. The cells were broken by crusher FastPrep-24TM5G and the cell lysis efficiency was checked under a microscopy. Once the cells were totally broken, the samples were incubated at −20°C for 30min and then centrifuged at 12,000*g* for 5min at 4°C. The supernatant was transferred to a new 1.5ml tube and stored at −80°C until further analysis. The protein concentrations of all samples were measured in parallel by Bradford assay. All experiments were repeated three times.

For quantification, the supernatants were evaporated and dried by vacuum manifold. The pellets were resuspended in an equal volume of Milli-Q-purified water. 20μl of each sample was analyzed by UPLC-MS/MS on ACQUITY UPLC H-class-Xevo TQ MS system, equipped with a Waters BEH C18 2.1×50mm column. All samples were filtered with a 0.22μm Ultra free-MC membrane before subjected to the column. The sample was separated at 25°C with a flow rate of 0.3ml/min and the chromatograph process used was as the following: gradient of solvent A (10mM tributylamine plus 15mM acetic acid in 97:3 water: methanol) to solvent B (methanol): t=0min; A-99%: B-1%, t=2.5min; A-80%: B-20%, t=7.0min; A-35%: B-65%, t=7.5min; A-5%:B-95%, t=9.01min; A-99%: B-1%, t=10min (end of gradient). The c-di-GMP was detected with electrospray ionization using multiple reaction monitoring in negative-ion mode at m/z 689.16→344.31. The parameters for mass spectrum were as the following: capillary voltage, 3.5kV; cone voltage, 50V; collision energy, 34V; source temperature, 110°C; desolvation temperature, 350°C; cone gas flow (nitrogen), 50l/h; desolvation gas flow (nitrogen), 800l/h; collision gas flow (nitrogen), 0.15ml/min; and multiplier voltage, 650V. Standard c-di-GMP (Biolog) at the concentrations of 500nM, 250nM, 125nM, 62.5nM, 31.25nM, 15.62nM, and 7.81nM were used to generate the standard curve for calculating the c-di-GMP concentration of each sample.

### Microscopy

The microscope images were captured by SDPTOP EX30 microscope. Microscope images taken at the indicated times and conditions were used for analyzing the heterocyst frequency and vegetative cell intervals. All experiments were analyzed with three parallel analyses.

### Quantitative Real-Time PCR

Wide-type *Anabaena* was cultivated to the log phase in BG11 and then transferred to BG11_0_ medium for heterocyst induction as described previously (Golden et al., 1991). To prepare the samples, 30ml of *Anabaena* were harvested at 0, 3, 16, 48, and 72h after nitrogen starvation, respectively, in triplicate. The total RNA was extracted with Plant Total RNA Isolation Kit (Foregene), and the genomic DNA was removed by treating with RNase-free DNase I (Promega, United States). The obtained total RNA samples were incubated with reverse transcriptase with HiScript Q RT super mix (Vazyme).

Quantitative Real-Time PCR was performed by using ChamQ SYBR qPCR Master Mix (Vazyme) with specific primers listed in Supplementary Table S3. The house keeping gene *rnpB* was used as an internal control. Primers Pallrs04F334 and Pallrs04R456 (for *rnpB*), PcdgS4F226 and PcdgSR402 (for *cdgS*), and PcdgSHF2342 and PcdgSHR2455 (for *cdgSH*), were used to amplify specific fragments. The RT-PCR products were confirmed by sequencing. Data were analyzed by ABI 7500 SDS software. To be consistent, the baseline was set automatically by the software. The comparative CT method (2^−△△CT^ method) was used to analyze the expression of *cdgS* and *cdgSH*. The data correspond to relative mRNA expression levels, shown as the mean±SE (standard error) of three replicates (*N*=3) analyzed by *t* test, and the significance was *p*<0.05.

### Protein Expression and Purification

The plasmids carrying recombinant genes for Strep-tag fusion proteins were transformed into competent cells of *E. coli* BL21 (DE3; Novagen) and then grew in LB medium with kanamycin (50μg/ml). When the cell culture reached OD_600_=0.5–0.6, 0.5mM IPTG was added and continued to grow overnight at 18°C. Cells were harvested by centrifugation at 8000*g* for 5min, resuspended in buffer W (100mM Tris-HCl pH 8.0, 1M NaCl and 1mM EDTA) and lysed by French press. The lysate was cleared by centrifugation at 12000*g* for 30min at 4°C, and the supernatant was incubated with buffer W equilibrated Strep-Tactin® XT Superflow® resin (IBA) at 4°C for 1h. The resin was then collected by filtration and washed with 10 column volumes (CV) of buffer W. The proteins were eluted with elution buffer (buffer W containing 50mM biotin) and dialyzed against 50mM Tris–HCl (pH 8.0), 10mM MgCl, 0.5mM EDTA, and 300mM NaCl for 12h at 4°C.

### *In vitro* Assays of DGC and PDE Activity

The DGC and PDE activity assay was analyzed as previously described (Enomoto et al., 2015). In brief, the total volume of each reaction was 50μl and the reaction mixture contained 50mM Tris·HCl pH 8.0, 10mM MgCl_2_, 0.5mM EDTA, 300mM NaCl, 50μM GTP (Thermo scientific) for DGC or 50μM c-di-GMP (Biolog) for PDE. Each reaction was initiated by adding the corresponding protein at a final concentration 2μM, and then incubated at 25°C for 30min. All the reactions were stopped by adding 20mM EDTA and immediately heated at 95°C for 5min. Before subjected to HPLC analysis, all samples were filtered with a 0.22μm Ultrafree-MC membrane by centrifugation. Nucleotides were separated by reverse-phase HPLC through a C18 column (150mm×6mm i.d.; DAISOPAK SP-120-5- ODS-AP; Daiso). 20μl sample from each reaction was injected and eluted with buffer A (50mM potassium phosphate, 4mM tetrabutylammonium hydrogen sulfate, pH 6.0) and buffer B (75% buffer A, 25% methanol, V/V) at 1.2mlmin^−1^, and the elution protocol as followed: 0min, 80%/20% A/B; 2.5min, 80%/20% A/B; 5min, 70%/30% A/B; 14min, 60%/40% A/B; 25min, 0%/100% A/B; 32min, 0%/100% A/B; 33min, 80%/20% A/B; and 34min, 80%/20% A/B. The nucleotides were detected at 254nm. Standard c-di-GMP and GTP at concentrations of 10, 20, 40, 80, 160, 320, and 640μM in the reaction buffer was used to generate the standard curve for quantifying the c-di-GMP and GTP levels, respectively, in reactions.

## Results

### c-di-GMP Level Increases Transiently During Heterocysts Development

**Figure 1:**
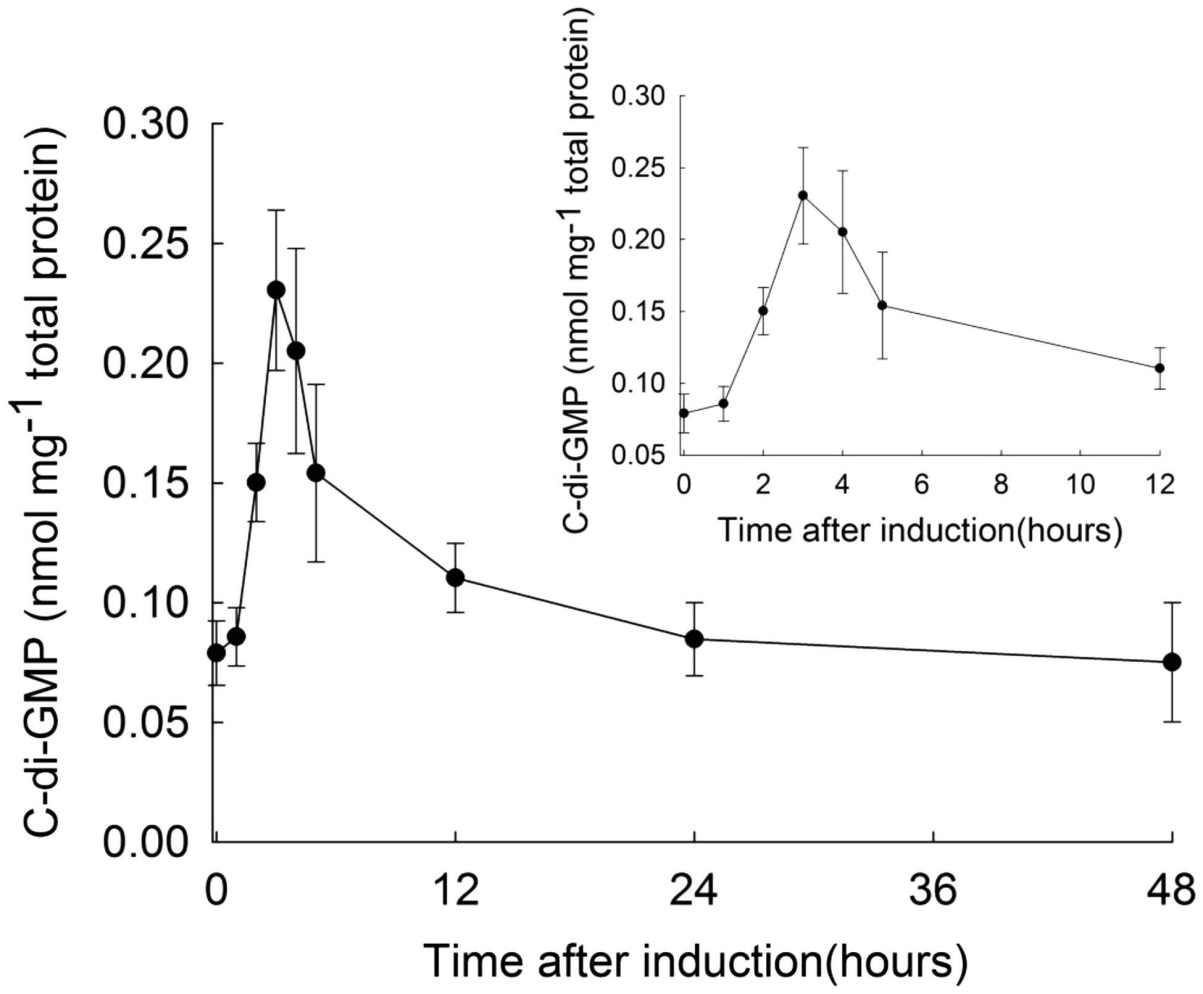
Intracellular c-di-GMP level of *Anabaena* at different time points during heterocyst development. *Anabaena* wild type cells were cultured in BG11 with nitrate, then transferred to BG11_0_ medium (without nitrate) for the initiation of heterocyst differentiation (time 0h). Cell samples were collected at different time points as indicated, and samples were prepared for c-di-GMP quantification by UPLC-MS/MS. The c-di-GMP concentration at the indicated time points was calculated according to the amount of total proteins of *Anabaena*. Standard deviation calculated from three biological replicates is shown. The inset shows details of the c-di-GMP levels from 0 to 12h after combined-nitrogen deprivation.

To investigate the relationship between c-di-GMP and heterocysts development, we first determined the changes of intracellular concentration of c-di-GMP during this physiological process in *Anabaena*. c-di-GMP from wild-type (WT) cultures at different time points after nitrogen starvation was extracted and quantified by ultra-high performance liquid chromatography with tandem mass spectrometry detection (UPLC-MS/MS). As shown in Figure 1, the c-di-GMP level in cells in BG11 medium (time 0) was 0.078±0.014nmol/mg protein. After exposure of cells to BG11_0_ medium, c-di-GMP levels increased by 3-fold at 3h after the transfer, reaching a peak level of 0.231±0.034nmol/mg protein. After this time point, the levels of c-di-GMP gradually decreased to the basal level in 24h. These results suggested that c-di-GMP may be an important signaling molecule during heterocyst differentiation.

### *ΔcdgSH* Mutant Caused an Increase in Heterocyst Frequency

**Figure 2:**
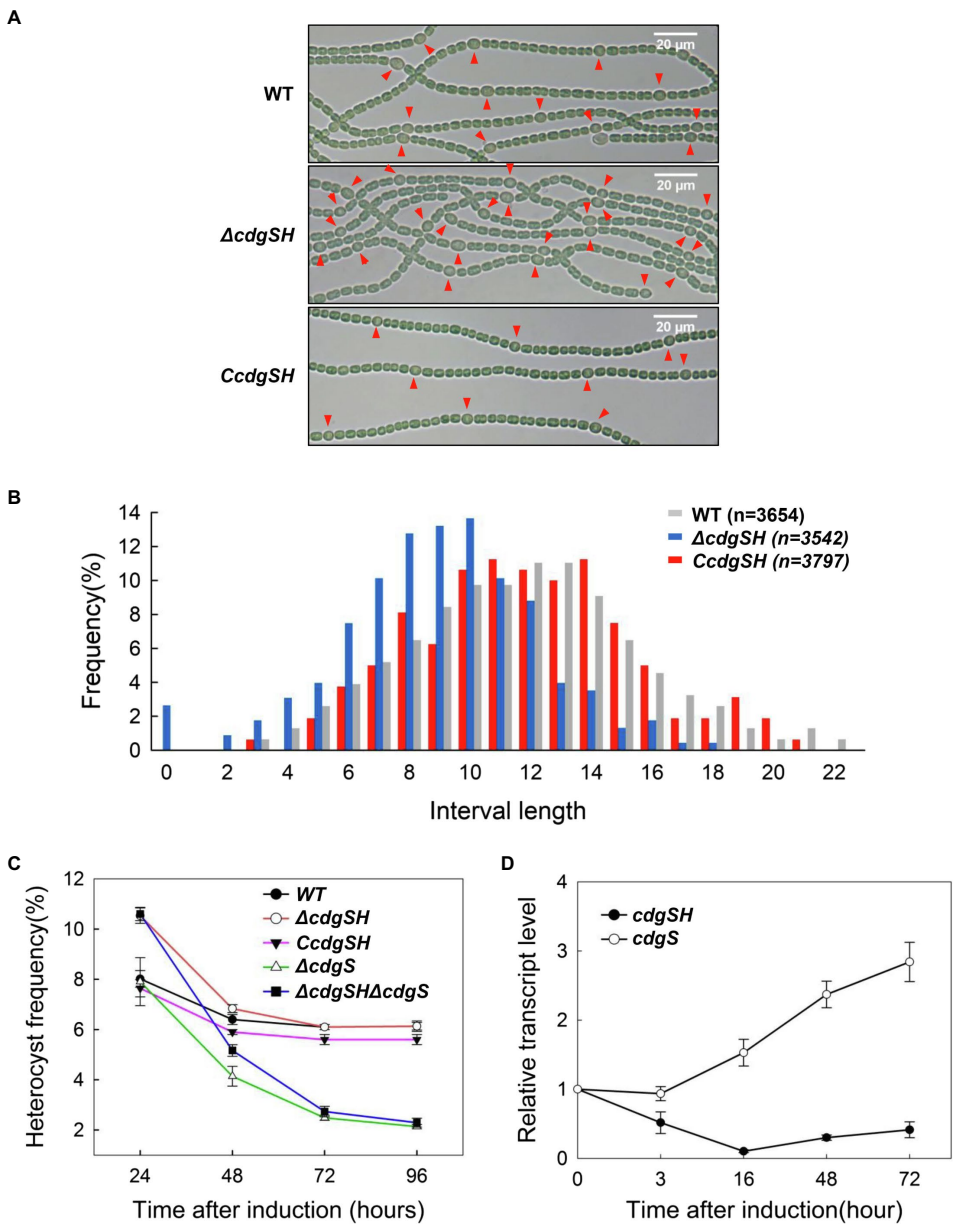
Deletion of 2 genes (*cdgSH* and *cdgS*) involved in c-di-GMP synthesis or hydrolysis affects heterocyst frequency. **(A)** Light microscopic images of filaments from wild type (WT), *cdgSH*-deletion strain (*ΔcdgSH*), and the complemented strain (C*cdgSH*, corresponding to *ΔcdgSH* carrying the *cdgSH* gene on a replicative plasmid), at 24h after the initiation of heterocyst differentiation induced by deprivation of combined nitrogen. Heterocysts are indicated by red triangle. **(B)** Analysis of heterocyst pattern in different strains as in **(A)** at 24h after initiation of heterocyst differentiation. The number of vegetative cells between two heterocysts (interval length) was counted, and the distribution of heterocyst intervals was analyzed according to the frequency of each interval. The number of the quantified vegetative cells is indicated in parentheses above the graph. **(C)** The heterocyst frequency of the indicated strains at different time points after nitrogen stepdown (24, 48, 72, and 96h). **(D)** The transcription levels of *cdgSH* and *cdgS* in WT. Samples were taken at the indicated time point after nitrogen stepdown, and total RNAs were extracted, and analyzed by quantitative PCR (qPCR). All the values are showed as mean±standard deviation, calculated from three biological replicates.

To investigate the role of c-di-GMP in heterocyst differentiation, in-frame markerless deletion of all 16 genes encoding proteins with predicted DGC and/or PDE domains were generated by genome editing technique based on CRISPR-Cpf1 (Supplementary Figure S1; Niu et al., 2019). We determined the ability of heterocyst formation for all mutants grown in BG11_0_. When observed under the microscope, 2 of the 16 mutants, *ΔcdgSH* and *ΔcdgS*, were affected in heterocyst frequency while all others had a heterocyst pattern and percentage comparable to those of the WT (Table 1). The phenotype of *ΔcdgS* was similar to that already reported (Neunuebel and Golden, 2008). Indeed, heterocyst frequency of the mutant was similar to that of WT at 24h after the induction (8.0±0.27%) and dropped by 60% (2.5±0.08%) 3days later after the induction as compared to the WT (6.2±0.16%). It can grow in BG11_0_, but less well than the WT (Supplementary Figure S6B). While *cdgS* was required to maintain heterocyst frequency over time, without affecting the initial heterocyst pattern *ΔcdgSH* showed a very different phenotype. After 24h of growth in BG11_0_ medium, *ΔcdgSH* mutant could differentiate mature heterocysts; however, the percentage of heterocysts showed an increase (10.3%±0.43%) when compared to WT (8.0%±0.33%), and a Mch (multiple-contiguous-heterocysts) phenotype was occasionally observed (Figures 2A,B). To confirm this phenotype, we further quantified the pattern of heterocysts along the filaments. Heterocyst intervals of WT show regularity, with 11 to 13 vegetative cells as the most representative intervals. In *ΔcdgSH* mutant, the distribution still kept a regularity, but the intervals shortened to 8 to 10 vegetative cells as the most representative ones (Figure 2B). Interestingly, from 48h after induction, the heterocyst frequency of the *ΔcdgSH* mutant became similar to WT (Figure 2C), indicating that the mutation affected only the initial establishment of the heterocyst frequency, but not its maintenance. The *ΔcdgSH* can grow well in BG11_0_ (Supplementary Figure S6A). The phenotype of *ΔcdgSH* could be complemented by *cdgSH* controlled by its own promoter present on a replicative plasmid (Figure 2C, strain C*cdgSH*). Taken together, among the 16 annotated genes related to c-di-GMP levels on the genome, *cdgSH* and *cdgS* appear to be the major ones involved in the regulation of heterocyst pattern.

**Table 1:** Heterocyst frequency of 16 mutants affecting c-di-GMP synthesis or hydrolysis at 24 and 72h after nitrogen starvation.

Strain	Heterocyst frequency 24h	Heterocyst frequency 72h
*WT*	8.0 ± 0.33%	6.2 ± 0.16%
*Δall1012*	7.8 ± 0.12%	5.8 ± 0.41%
*Δall1219*	7.9 ± 0.30%	6.1 ± 0.31%
*Δall2416*	7.8 ± 0.24%	5.9 ± 0.43%
*Δall2874 (cdgS)*	8.0 ± 0.27%	2.5 ± 0.08%
*Δalr3504*	8.1 ± 0.46%	5.6 ± 0.56%
*Δalr3599*	8.3 ± 0.16%	6.1 ± 0.32%
*Δall4896*	8.1 ± 0.41%	5.7 ± 0.18%
*Δall5174*	7.7 ± 0.19%	6.1 ± 0.42%
*Δalr1230*	8.2 ± 0.54%	6.2 ± 0.18%
*Δall0219*	8.0 ± 0.37%	6.1 ± 0.38%
*Δall1175 (cdgSH)*	10.5 ± 0.43%	6.0 ± 0.27%
*Δall4225*	7.6 ± 0.22%	6.2 ± 0.33%
*Δall4897*	8.2 ± 0.11%	5.6 ± 0.24%
*Δalr2306*	8.1 ± 0.29%	5.8 ± 0.28%
*Δalr3170*	8.1 ± 0.42%	6.0 ± 0.32%
*Δalr3920*	8.3 ± 0.03%	6.2 ± 0.21%

### CdgSH and CdgS Have Additive Effect for Regulating Heterocyst Frequency

Although both *cdgSH* and *cdgS* regulate heterocyst intervals, their phenotypes were very distinct. To investigate the relationship between *cdgSH* and *cdgS* in regulating heterocyst intervals, we constructed a *ΔcdgSHΔcdgS* double mutant. 24h following combined-nitrogen deprivation, heterocyst frequency of this double mutant was 10.5%±0.21%, similar as *ΔcdgSH*. However, with prolonged time in combined-nitrogen free medium, the frequency decreased gradually, and reaching 5.2%±0.19, 2.5%±0.17 and 2.1%±0.14% at 48, 72, and 96h, respectively (Figure 2C), a phenotype similar as *ΔcdgS*. Although the two single mutants displayed opposite effect on the frequency of heterocysts, the results indicated that the phenotype of the double mutant was the sum of the single ones under diazotrophic condition. This indicated that these two genes acted independently at different time points during heterocyst development, with *cdgSH* for establishment of heterocyst pattern (the first 24h), while *cdgS* for maintenance of the frequency of heterocysts at the later stages.

Next, to better understand how the two genes could participate at different time points in heterocyst development, we checked the transcription levels of *cdgSH* and *cdgS* in WT at different time points after nitrogen starvation by quantitative Real-Time PCR. Our data indicated that these two genes showed opposite transcriptional profiles (Figure 2D). Following nitrogen stepdown, the transcript level of *cdgS* was kept steady in the first 3h, and then increased after 16h post induction. At 72h after combined-nitrogen deprivation, the relative transcript level of *cdgS* was increased around 3-fold. In contrast, under the same conditions, the transcriptional activity of *cdgSH* was dropped to nearly undetectable level at 16h after transfer to BG11_0_. Although gradually increased after 16h, it still remained at a much lower at 72h as compared to that observed at time 0 (Figure 2D). The transcriptional profiles of these two genes are in good agreement with the phenotype of their corresponding mutants. Indeed, *cdgSH* had a higher transcriptional activity at the onset of heterocyst induction, and its inactivation affected the establishment of heterocyst frequency. In contrast, the transcriptional activity of *cdgS* was low at the beginning of heterocyst induction, but increased steadily afterwards; consistently, heterocyst frequency continuously dropped from the second day overtime after combined-nitrogen deprivation.

### CdgSH Is a Bi-Functional Enzyme With a Dominating PDE Activity *in vitro*

**Figure 3:**
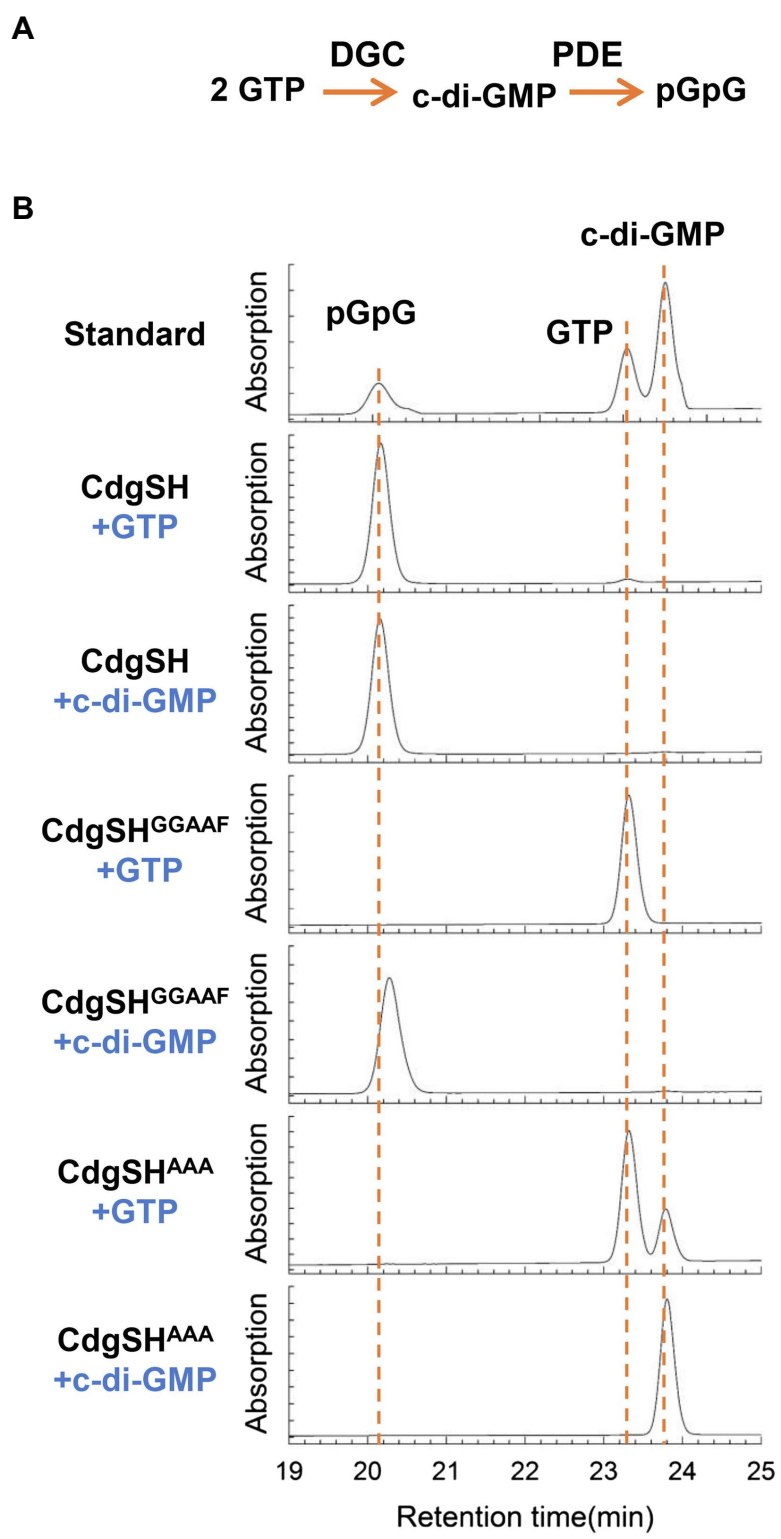
*Anabaena* protein CdgSH has both diguanylate cyclase (DGC) and c-di-GMP-specific phosphodiesterase (PDE) activities. **(A)** Scheme showing the reactions catalyzed by the PDE (c-di-GMP→pGpG) and DGC (2 GTP→c-di-GMP) activities of an enzyme. **(B)** The enzymatic activities of CdgSH and its variants CdgSH^GGAAF^ (inactivating the DGC activity) and CdgSH^AAA^ (abolishing the PDE activity) were evaluated by the reaction products. Recombinant proteins were produced from *E. coli*, purified, and tested. All reaction substrates or products were assessed by the retention time using HPLC according to the standards. The top panel shows the retention time of the standard nucleotides: pGpG, c-di-GMP, and GTP. The retention time of each nucleotide was first determined by HPLC separately (data not shown here). The proteins used for PDE and DGC activity assay were indicated at the left of the chromatograms, and the substrate for each reaction was indicated under the name of proteins. CdgSH can convert substrates, GTP and c-di-GMP, to pGpG. PDE active-site mutant protein CdgSH^AAA^ can only convert GTP to c-di-GMP, and the CdgSH^GGAAF^, which inactivates the DGC activity of CdgSH, can only hydrolyze c-di-GMP to pGpG.

CdgSH is predicted to be a multi-domain cytoplasmic protein, with FHA, PAS, GAF, GGDEF and EAL domains arranged in tandem (Supplementary Figure S1). The GGDEF and the EAL domains are both located towards the C-terminus of the polypeptide and contain all the conserved residues required for their corresponding catalytic activity (Supplementary Figure S2). This observation suggests that CdgSH may exhibit both c-di-GMP synthesis and degradation activities. To check this observation, CdgSH and its variants were expressed in *E. coli* BL21 (DE3) and purified using a strep-tag affinity column (Supplementary Figure S3). The DGC and PDE activities of these proteins were then determined. Our results showed that no matter which of the substrates, GTP or c-di-GMP, was used, only one product, pGpG, could be detected by HPLC (Figure 3). This indicated that CdgSH could synthesize c-di-GMP from GTP, and immediately hydrolyzed c-di-GMP to pGpG. To further confirm this observation, the CdgSH^GGAAF^ variant, which the catalytic and the metal ion coordination sites were replaced (mutations D530A and E531A), was produced (Chan et al., 2004; Wassmann et al., 2007). This variant kept an intact PDE domain, but a defective DGC domain. As shown in Figure 3, CdgSH^GGAAF^ could only degrade c-di-GMP to pGpG, and when GTP was added, no activity was detected. The CdgSH^AAA^ variant, which inactivated the PDE activity of CdgSH by mutating the metal ion coordination sites (mutations E656A and L658A; Barends et al., 2009; Tchigvintsev et al., 2010), was also tested, and it could synthesize c-di-GMP from GTP, but the c-di-GMP hydrolysis activity was abolished (Figure 3).

To further investigate the relationship between the PDE and DGC activities of CdgSH, we quantified the steady-state kinetic parameters (*kcat* and *Km*) for CdgSH, CdgSH^GAAEF^ and CdgSH^AAA^ under the same experimental conditions. As shown in Table 2, the *kcat* value for the PDE activity is three times that of the DGC activity in CdgSH, while the DGC *Km* values is 1.5 times that of the PDE activity. This result indicates that although the binding efficiencies of the two catalytic domains to their respective substrate are similar, the conversion efficiencies are quite different. Indeed, the catalytic activity of PDE in CdgSH, expressed by the *kcat/Km* ratio is 5.4×10^−2^μM/S^−1^ (Table 2), which is more than 5-fold higher than that of DGC (1.0×10^−2^μM/S^−1^). In addition, we found that the catalytic activity (*kcat*/*Km*) of PDE in CdgSH and CdgSH^GGAAF^ are similar to each other, suggesting that the PDE activity of CdgSH was independent of the DGC activity. On the other hand, the catalytic activity (*kcat*/*Km*) of DGC in CdgSH^AAA^ decreased 3-fold when compared to that of CdgSH, suggesting the existence of a feedback inhibition of the DGC activity by c-di-GMP. Such a phenomenon was known for typical GGDEF domains, which can be explained by the presence of the conserved inhibitory site (RXXD motif) present in CdgSH which allows c-di-GMP binding and allosteric feedback inhibition (Supplementary Figure S2; Chan et al., 2004; Wassmann et al., 2007). Taken all together, our results demonstrated that CdgSH was a bi-functional enzyme with both DGC and PDE activities, but its PDE activity dominated over the DGC activity, making the c-di-GMP hydrolysis a prevailing output at the end.

**Table 2:** The steady-state kinetics of CdgSH and its variants CdgSH^GGAAF^ (abolishing the synthesis activity) and CdgSH^AAA^ (abolishing the hydrolysis activity).

Enzyme	DGC (GTP→c-di-GMP)			PDE (c-di-GMP→pGpG)			Km (μM)	Kcat (s^−1^)	Kcat/Km (μMs^−1^)	Km (μM)	Kcat (s^−1^)	Kcat/Km (μMs^−1^)
CdgSH	37.2	0.39	1.05×10^−2^	23.5	1.26	5.4×10^−2^
CdgSH^GGAAF^	—	—	—	31.0	1.78	5.7×10^−2^
CdgSH^AAA^	34.8	0.12	3.45×10^−3^	—	—	—

For comparison, we also tested the enzymatic activity of CdgS which possesses an N-terminal REC domain and a C-terminal GGDEF domain under the same experimental conditions (Supplementary Figures S1, S2). Our results indicated that CdgS could convert the substrate GTP to c-di-GMP (Supplementary Figure S4), consistent with the result already published (Neunuebel and Golden, 2008). When the catalytic site and the metal ion coordination site (E229A, and E230A) of DGC was replaced (Chan et al., 2004; Wassmann et al., 2007), the c-di-GMP synthesis activity of CdgS^GGAAF^ was completely abolished (Supplementary Figure S4). Therefore, CdgS is a typical c-di-GMP synthase.

### *ΔcdgSH* Mutant Phenotype Can Be Complemented by Expression of a Heterologous PDE-Encoding Gene, but Not a Heterologous DGC-Encoding Gene

**Figure 4:**
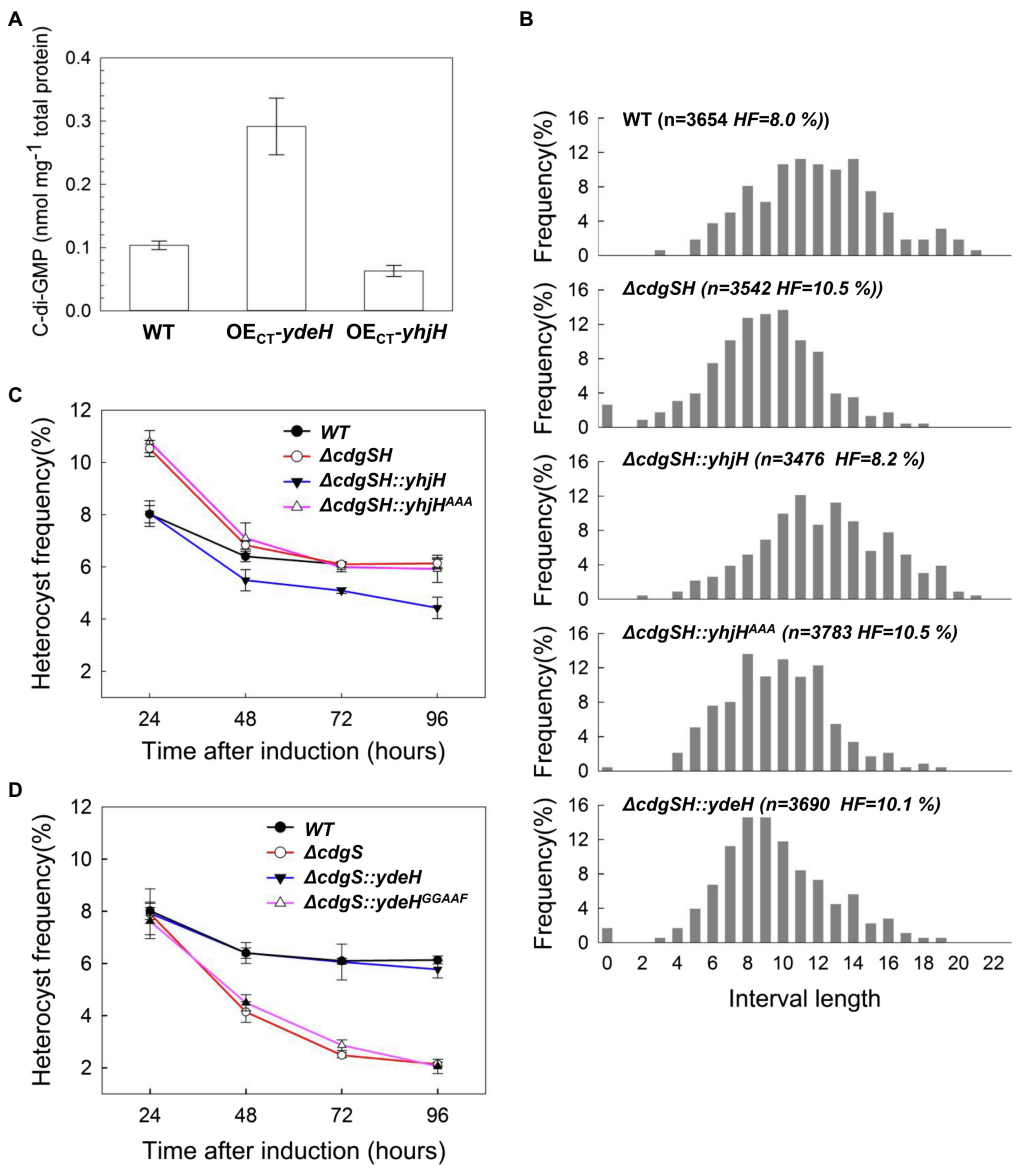
The phenotype of *ΔcdgSH and ΔcdgS* can be complemented by expression of a heterologous PDE (*yhjH*) and a heterologous DGC (*ydeH*), respectively. **(A)** The intracellular c-di-GMP level of the indicated strains, measured at 24h after the addition of the inducers (0.5μM copper and 2mM theophylline). OE_CT_-*ydeH*, a strain derived from the WT expressing *ydeH* from *E. coli* under the control of the CT promoter inducible by copper and theophylline; OE_CT_-*yhjH*, a WT strain expressing yhjH from *E. coli* under the control of the CT promoter. All the values of c-di-GMP concentration are expressed as mean±standard deviation, calculated from three replicates. **(B)** Distribution of heterocysts along the filaments analyzed as heterocyst intervals at 24h after nitrogen starvation. Interval length represents number of vegetative cells between two heterocysts. Heterocyst frequency (HF) and number of vegetative cells counted at this time point was indicated in parentheses. *ΔcdgSH*::*yhjH*, the *ΔcdgSH* mutant complemented by *yhjH*, a gene from *E. coli* encoding a PDE, under the control of the *cdgSH* promoter; *ΔcdgSH*::*yhjH^AAA^* correspond to *ΔcdgSH* carrying *yhjH* in which the active site EAL was mutated to AAA; *ΔcdgSH*::*ydeH* is *ΔcdgSH* with the expression of *ydeH* (encoding a DGC enzyme) from *E. coli* under the control of the *cdgSH* promoter. **(C,D)** The heterocyst frequency of the indicated strains at different time points after nitrogen stepdown (24h, 48h, 72h, and 96h). All the values of heterocyst frequency are showed as mean±standard deviation, calculated from three replicates. In **(D)**, the strain *ΔcdgSH*::*ydeH^GGAAF^* is similar to *ΔcdgSH*::*ydeH* except the active site of the DGC activity, GGDEF was mutated to GGAAF.

CdgSH is a protein containing other domains in addition to the two with opposing activities in c-di-GMP synthesis and hydrolysis. To evaluate the contribution of the different activities of CdgSH in regulating heterocyst frequency, we examined the ability of heterologous genes encoding c-di-GMP metabolic enzymes to complement the *ΔcdgSH* mutant. YdeH (DGC) and YhjH (PDE) are well-studied c-di-GMP metabolic enzymes from *E. coli* with high catalytic activity *in vitro* (Pesavento et al., 2008). Each of the two proteins contains only one catalytic domain, without additional regulatory domains (Supplementary Figure S2). Therefore, they are often used to alter the intracellular c-di-GMP level of various bacteria (Tschowri et al., 2014; Li et al., 2018). To examine their activities in *Anabaena*, plasmids containing *ydeH* or *yhjH* under the control of inducible regulatory elements (the CT promoter) was transferred into *Anabaena* by conjugation, giving the strain OE_CT_-*ydeH* or OE_CT_-*yhjH*, respectively. We then determined the intracellular c-di-GMP levels of the two recombinant stains. Our data revealed that the intracellular c-di-GMP level of the two recombinant strains, growing in BG11 medium and induced by adding 2mM theophylline and 0.5μM Cu^2+^, displayed significant changes as compared with WT after 24h of induction. The c-di-GMP level of the cells in OE_CT_-*ydeH* was at 0.29±0.04nmol/mg total protein, corresponding to an increase by 2-fold relative to that of WT. Under similar conditions, cells of OE_CT_-*yhjH* decreased the level of c-di-GMP by 2-fold, to 0.06±0.01nmol/mg total protein (Figure 4A). Therefore, YdeH and YhjH are active in *Anabaena*, and their production could modify the intracellular levels of c-di-GMP in this organism.

Next, we analyzed the heterocyst pattern and frequency of the strains expressing *yhjH* or *ydeH* in the *ΔcdgSH* background. To make the data comparable, we used the native promoter of *cdgSH* to drive the expression of *yhjH* or *ydeH* on a replicative plasmid. We found the heterocyst pattern in the recombinant strain *ΔcdgSH*::*yhjH* was restored to the WT pattern, with 11 to 13 vegetative cells as the most representative intervals after 24h combined nitrogen step-down and the heterocyst frequency at this time point was also restored to the WT level, to about 8.2±0.35% (Figure 4B). In contrast, the strain *ΔcdgSH*::*yhjH^AAA^* bearing the same plasmid but containing the mutant allele of *yhjH* (*yhjH^AAA^*) failed to complement the defect in heterocyst distribution and frequency of *ΔcdgSH* (Figure 4B). Furthermore, the -strain *ΔcdgSH*::*ydeH* was unable to restore the change in heterocyst distribution and frequency of the *ΔcdgSH* mutant (Figure 4B; Supplementary Figure S3). Altogether, our results indicated that the c-di-GMP hydrolysis activity of CdgSH, and hence the decrease in c-di-GMP level, was the major element that contributes to the regulation of heterocyst frequency of *ΔcdgSH* mutant at 24h after nitrogen starvation. With prolonged time in combined-nitrogen free medium, *ΔcdgSH:: yhjH* strain had a slightly lower heterocyst frequency as compared to the WT from 48 to 96h after the transfer to BG11_0_ medium (Figure 4C). This phenotype is similar to *ΔcdgS* mutant which also displayed a drop in heterocyst frequency as compared to the WT at the same time points. This result could be explained by the fact that the gene yhjH in the complemented strain was carried on a replicative plasmid that is known to lead to a higher level expression than that from the chromosome, and also the fact that YhjH possess a high PDE activity.

We also tested whether *ydeH* could complement *ΔcdgS*. Indeed, the *ydeH* gene under the control of the native promoter of *cdgS*, carried on a replicative plasmid could complement *ΔcdgS* successfully (strain *ΔcdgS::ydeH*, Figure 4D). *ydeH^GGAAF^* which encodes an inactive form of YdeH (E208 and E209, Figure 4D) was unable to complement the phenotype of *ΔcdgS* mutant when controlled by the same regulatory system (Figure 4D). These studies indicated that the c-di-GMP metabolic activities of CdgSH and CdgS were the major reason for the phenotype observed following the inactivation the corresponding genes in *Anabaena*.

### Homeostasis of c-di-GMP Is Critical for Heterocyst Differentiation

**Figure 5:**
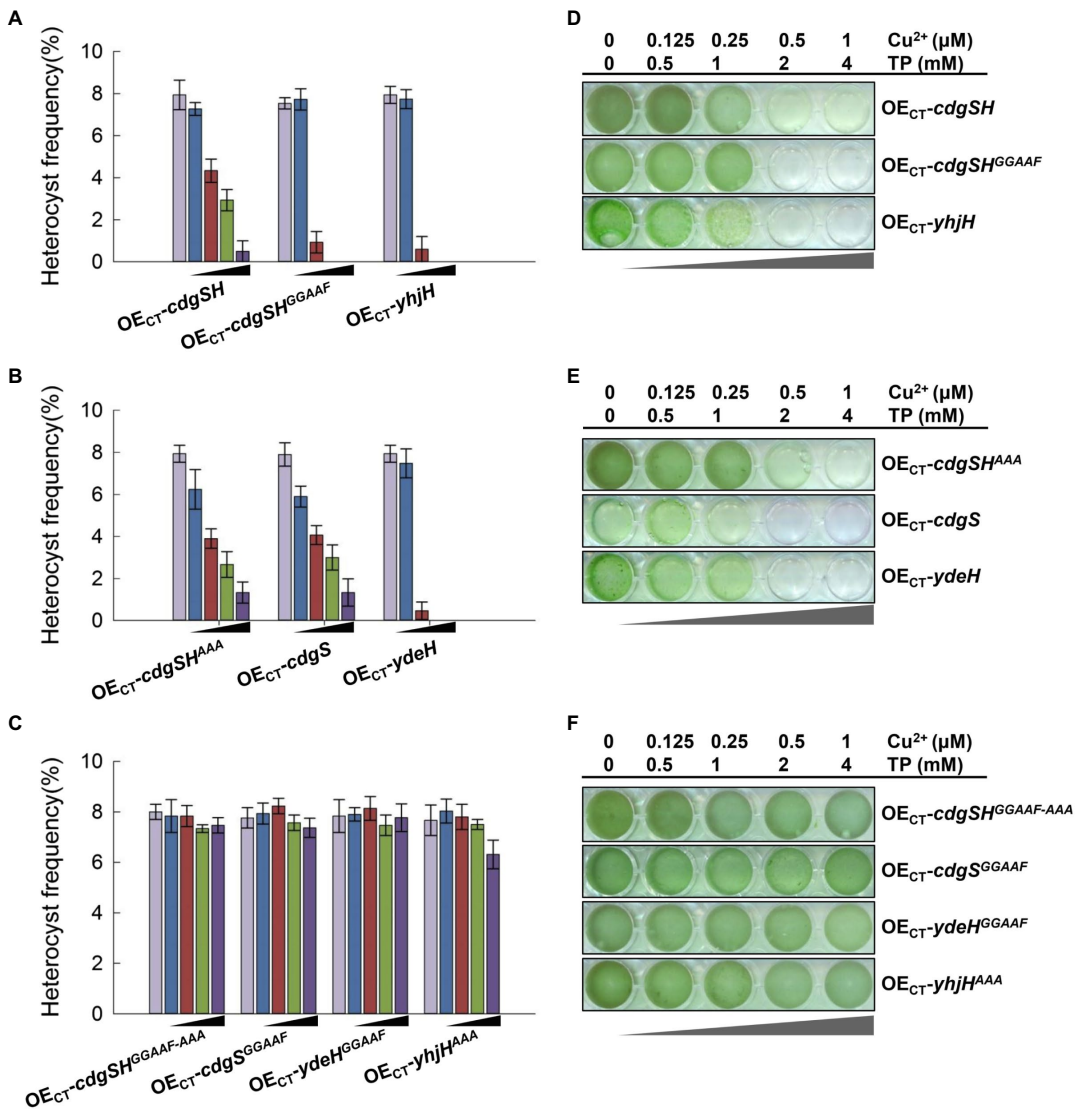
High levels ectopic inductions of PDE or DGC activities inhibited heterocyst development. **(A–C)** Heterocyst frequency of the indicated overexpression strains at 24h after nitrogen stepdown with five concentrations of inducers. The black triangle indicated the increasing levels of the inducers added to the culture media (from left to right column): 0μM copper and 0mM theophylline, 0.125μM copper and 0.5mM theophylline, 0.25μM copper and 1mM theophylline, 0.5μM copper and 2mM theophylline, and 1μM copper and 4mM theophylline. **(A)** The ectopic overexpression of enzymes with PDE activity, including *Anabaena* CdgSH (strain OE_CT_-*cdgSH*), CdgSH variants that abolished the DGC activity (strain OE_CT_-*cdgSH^GGAAF^*) and YhjH from *E. coli* (strain OE_CT_-*yhjH*). **(B)** The overexpression strains producing enzymes with DGC activity, including *Anabaena* CdgS (strain OE_CT_-*cdgS*), CdgSH variants with the PDE active site mutated (strain OE_CT_-*cdgSH^AAA^*), and YdeH from *E. coli* (strain OE_CT_-*ydeH*). **(C)** The overexpression strains with production of enzymes that have no active PDE and/or DGC activity, including the *Anabaena* CdgSH variant in which both the DGC active site and the PDE active site were mutated (strain OE_CT_-*cdgSH^GGAAF-AAA^*), the CdgS variant abolishing the DGC active site (strain OE_CT_-*cdgS^GGAAF^*), the *E. coli* YhjH variant with the EAL active site changed to AAA (strain OE_CT_-*yhjH^AAA^*), and the *E. coli* YdeH variant with the GGDEF active site changed to GGAAF (strain OE_CT_-*ydeH^GGAAF^*). **(D–F)** The growth of different strains as in **(A–C)** tested in 24-well plates. All cultures were started with a similar OD at 0.3 diluted from a pre-culture and imaged after 4days of incubation.

The metabolism of c-di-GMP should be highly regulated during heterocyst development, as demonstrated by the changes of the c-di-GMP levels during the developmental process, as well as the functions of the two genes *cdgSH* and *cdgS*, acting at different phases of heterocyst differentiation. To check whether such regulation is important, we examined the effect caused by varying the intracellular levels of c-di-GMP. For this purpose, CdgSH, CdgS, and their corresponding mutant variants were expressed in WT cells of *Anabaena*, respectively. The expression levels of the genes were titrated by a gradient of inducers as shown in Figure 5 and heterocyst development was observed at 24h after the initiation by transfer to BG11_0_. When *cdgSH* was tested with increasing concentrations of inducers, heterocyst frequency gradually decreased from 8 to 1.3%. A more pronounced results were obtained when CdgSH^GGAAF^ in which the activity of DGC was abolished, since at the highest concentrations of inducers tested, few heterocysts were formed (0.5%; Figure 5A). These results were consistent with the fact that CdgSH has both c-di-GMP synthesis and hydrolysis activities, but the hydrolysis activity was dominant, while the CdgSH^GGAAF^ variant had only the PDE activity left (Figure 3; Table 2).

Interestingly, adding increasing gradients of inducers to express either the CdgSH^AAA^ variant that has only retained the activity of c-di-GMP synthesis or CdgS also led to a reduction of heterocyst frequency. At 24h after combined-nitrogen deprivation, with 4mM theophylline and 1μM Cu^2+^, the heterocyst frequency of both strains was around 1%, while heterocyst frequency is 8% without inducers (Figure 5B). Therefore, ectopic expression of genes encoding either c-di-GMP synthase or hydrolase activities repressed heterocyst differentiation.

To further confirm it is the c-di-GMP metabolic activities of CdgSH that was responsible for such phenotypes, we tested the strain of OE_CT_-*ydeH* or OE_CT_-*yhjH* at the same condition, respectively. With no inducers or with low concentrations of 0.5mM theophylline and 0.125μM Cu^2+^, the heterocysts of YdeH and YhjH overexpression strains were formed with a frequency similar as the WT (Figures 5A,B). However, when the concentrations of inducers increased to 1mM for theophylline and 0.25μM for Cu^2+^, the heterocysts frequency of both strains were reduced to about 1% at 24h after the initiation. Heterocyst developments of both strains were completely suppressed when the concentration of the inducers reached for 2mM theophylline and 0.5μM Cu^2+^ (Figures 5A,B). These results were consistent with those obtained by the ectopic expression of the CdgSH, CdgSH variants and CdgS. To exclude the influence of high dose of inducers or proteins *in vivo*, effects of production of CdgSH^GGAAF-AAA^, CdgS^GGAAF,^ YdeH^GGAAF^_,_ and YhjH^AAA^ variants, which c-di-GMP synthesis or degradation activities were abolished, as well as WT strain were also examined under the same experimental conditions (Figure 5C; Supplementary Figure S5A). Even at the highest concentrations of the two inducers added, heterocyst frequency and pattern were hardly affected.

We also compared the growth capacity of different strains under diazotrophic conditions. As the proportion of heterocysts decreased, the diazotrophic growth capacity of the strains decreased as well (Figures 5D–F; Supplementary Figure S5B). The growth capacity of control strains remained similar under different inducer conditions. Taken together, our results showed that depending on the promoter used (native promoters of *cdgSH* or *cdgS*, or the synthetic CT promoter), and the concentrations of the inducers employed, expression of the synthase or hydrolase activity of c-di-GMP may have different outcomes. When expressed under the ectopic promoter (CT promoter) to a high level, both the hydrolase activity and the synthase activity could lead to a repressive effect on heterocyst differentiation, suggesting that intracellular c-di-GMP homeostasis in *Anabaena* is important for heterocyst development.

## Discussion

Heterocyst differentiation in cyanobacteria is regulated by multiple signals (Zhang et al., 2006). In this study, we provide evidence that the second messenger c-di-GMP constitutes a new signal for heterocyst differentiation, as already suggested by a previous study (Neunuebel and Golden, 2008). First, the level of c-di-GMP increases transiently following the initiation of heterocyst differentiation by deprivation of combined nitrogen (Figure 1). This accumulation profile is similar to, but lags behind, that of 2-oxoglutarate, a trigger of heterocyst formation (Laurent et al., 2005). It has been proposed that CdgS acts upstream of HetR, the master regulator of heterocyst development, and is required for the up regulation of *patS* encoding an inhibitor of heterocyst differentiation (Huang et al., 2004). The time window where the dynamic changes in the level of c-di-GMP was observed in this study is consistent with the early action of this second messenger in heterocyst development.

Secondly, when the c-di-GMP levels are modified by ectopic expression of two well-characterized genes from *E. coli*, *ydeH*, and *yhjH*, which could synthesize or hydrolyze c-di-GMP, respectively (Pesavento et al., 2008), heterocyst formation was inhibited (Figure 5). Therefore, the regulated change in the intracellular levels of c-di-GMP, and thus its homeostasis, is critical for heterocyst formation. However, when each of the genes involved in c-di-GMP metabolism was inactivated, none of the mutants produced such a strong phenotype. Genetic analysis of the genes revealed that at the single gene level, the role of c-di-GMP signal in heterocyst development was mostly attributed to the functions of the c-di-GMP synthetase CdgS and c-di-GMP hydrolase CdgSH (Figures 3, 4; Tables 1 and 2). The phenotypes of the two-corresponding mutant *ΔcdgSH* and *ΔcdgS* indicated the c-di-GMP regulates heterocyst frequency at different stages of the developmental process (Figure 2). Therefore, it is likely that at least some of the 16 genes encoding c-di-GMP synthesis or hydrolysis activities may have redundant functions in regulating heterocyst frequency. Such genetic redundancy may also partly account for the dynamic changes in the levels of c-di-GMP during heterocyst development, which cannot be fully explained by the action of CdgS and CdgSH alone. Indeed, following combined-nitrogen deprivation, it takes about 3h for c-di-GMP to climb to the peak level, then to decrease gradually to the basal level (Figure 1). At the transcriptional level, the amount of *cdgS* transcript detected (encoding a c-di-GMP synthesis enzyme) remains stable at 3h post induction, but that of *cdgSH* (encoding a dominant hydrolase activity of c-di-GMP) decreases (Figure 2). The combined effect of their expression is consistent with the increase in c-di-GMP level observed at the same time point. However, after 3h, the expression level of *cdgS* starts to increase, while that of *cdgSH* remains at a low level (Figure 2), which together should lead to an overall increase in c-di-GMP levels rather than a decrease as shown in Figure 1. Thus, although our genetic data indicate that CdgSH and CdgS appear to be the major individual players in regulating heterocyst frequency, other c-di-GMP metabolic enzymes must play important roles as well. Mutant strains, in which all c-di-GMP synthesis or degradation activities could be removed, respectively, would help us to elucidate the essential function of c-di-GMP in regulating heterocyst formation. In addition, an assessment of cell-type specific transcription of genes encoding all c-di-GMP metabolic enzymes would be necessary for a better understanding of their function in heterocyst development. In addition, as discussed below, these enzymes are also regulated at the enzymatic level too, which may also contribute to the dynamic changes of c-di-GMP under different environmental conditions.

Previously, systematic inactivation of 14 genes involved in c-di-GMP turnover was reported, which led to the identification of *cdgS*. In this study, systematic markerless deletion of all 16 genes by using the genome editing technique based on CRISPR-Cpf1 led to the identification of a new gene, *cdgSH*, required for heterocyst frequency (Neunuebel and Golden, 2008). The CRISPR-Cpf1 system allowed us to delete with efficiency a gene from a chromosome without leaving an antibiotic-resistance marker, so that the phenotype of the mutant could be analyzed without adding any antibiotic in the growth medium. The presence of a selective agent may affect phenotypic analysis, in particular when the phenotype is weak, such as that exhibited by the *cdgSH* mutant. Markerless deletion also simplified the deletion of multiple genes from the chromosome one after another with no limitation imposed by the use of selective antibiotics, and it could in addition avoid the polar effect caused to the downstream genes (Niu et al., 2019).

The C-terminal region of CdgSH has both a GGDEF and an EAL domain. Consistent with its domain structure, our biochemical data *in vitro* shows that it has the cyclase activity for c-di-GMP synthesis as well as the hydrolase activity for c-di-GMP degradation (Figure 3). Although the substrate binding efficiencies for both domains are similar, the catalytic activity of the hydrolase domain is more efficient than that of the cyclase domain, leading to c-di-GMP hydrolysis as the net outcome of the CdgSH activity *in vitro*. In addition, a feedback inhibition of c-di-GMP synthetase, a characteristic of all c-di-GMP cyclase with the presence the auto inhibition site (Chan et al., 2004; Wassmann et al., 2007), also revealed in CdgSH. The N terminal part of CdgSH possesses three sensor domains (FHA, PAS, and GAF); therefore, c-di-GMP turnover due to the activities of CdgSH should be highly regulated by different input signals acting on these sensor domains. Many PAS and GAF domains in bacteria are light or oxygen sensors due to their binding capacity of pigments, such as flavin mononucleotide, heme, phycocyanobilin, biliverdin, and so on (Christie et al., 1999; Gilles-Gonzalez and Gonzalez, 2004; Savakis et al., 2012; Enomoto et al., 2014; Narikawa et al., 2015). However, the PAS and GAF domains in CdgSH did not contain those conserved residues for pigment binding. Therefore, the signals that regulate the activities of CdgSH remain to be uncovered. In comparison, CdgS functions differently since it has only the GGDEF domain and thus the cyclase activity for c-di-GMP synthesis. CdgS has a putative response regulator domain and may also be controlled by signals that need to be identified (Supplementary Figure S1).

Overall, our genetic studies of the two genes in c-di-GMP metabolism, and the biochemical characterization of the corresponding enzymes, together with the use of heterologous enzymes from *E. coli*, all point out that the function of the two proteins in regulating heterocyst frequency is mediated by their enzymatic activity in c-di-GMP turnover. These results pave the way for further understanding the signaling function of c-di-GMP in *Anabaena*. It should be noted that although *Anabaena* has a large number of genes encoding proteins for c-di-GMP synthesis or degradation, no c-di-GMP receptors based on sequence similarity to those already known in other bacteria have been identified in this organism. Therefore, c-di-GMP signaling in cyanobacteria must have distinct features, and their identification will be a key for our understanding the function of c-di-GMP in the regulation of cyanobacterial physiology.

## Data Availability Statement

The original contributions presented in the study are included in the article/**Supplementary Material**, further inquiries can be directed to the corresponding author.

## Author Contributions

C-CZ, XZ, and J-YZ designed the research. MH performed the research. MH, XZ, and C-CZ analyzed the data and wrote the manuscript. All authors contributed to the article and approved the submitted version.

## Funding

This work was supported by Youth Program of National Natural Science Foundation of China (Grant No. 31800033), National Natural Science Foundation of China (Grant No. 92051106), the Key Research Program of Frontier Sciences of the Chinese Academy of Sciences (Grant No. QYZDJ-SSW-SMC016), and the Featured Institute Service Projects from the Institute of Hydrobiology, the Chinese Academy of Sciences (Grant Nos. Y85Z061601 and Y65Z021501).

## Conflict of Interest

The authors declare that the research was conducted in the absence of any commercial or financial relationships that could be construed as a potential conflict of interest.

## Publisher’s Note

All claims expressed in this article are solely those of the authors and do not necessarily represent those of their affiliated organizations, or those of the publisher, the editors and the reviewers. Any product that may be evaluated in this article, or claim that may be made by its manufacturer, is not guaranteed or endorsed by the publisher.
