# Supplementary material for: c-di-GMP Homeostasis Is Critical for Heterocyst Development in Anabaena sp. PCC 7120
Source: Front Microbiol. 2021 Dec 3;12:793336. doi: 10.3389/fmicb.2021.793336 (PMC8682488; doi:10.3389/fmicb.2021.793336)
Supplement: Supplementary file 1 [file Data_Sheet_1.DOCX]

SUPPLEMENTAL MATERIALS

**Table S1. Strains used in this study**

| **Strains** | **Description** | **Source** |
| --- | --- | --- |
| ***Escherichia coli*** |  |  |
| DH5α | Used for routine transformation |  |
| BL21 (DE3) | Used for protein expression |  |
| Alice | Used for conjugation | (ElhaI *et al*., 1997) |
| ***Anabaena* sp. strains** |  |  |
| PCC7120 | Wild type | Pasteur Culture collection |
| *Δall1012* | A markerless deletion mutant by removing the ORF of *all1012* | This study |
| *Δall1219* | A markerless deletion mutant by removing the ORF of *all1219* | This study |
| *Δall2416* | A markerless deletion mutant by removing the ORF of *all2416* | This study |
| *Δall2874* (*ΔcdgS)* | A markerless deletion mutant by removing the ORF of *all2874* | This study |
| *Δalr3504* | A markerless deletion mutant by removing the ORF of *alr3504*. | This study |
| *Δalr3599* | A markerless deletion mutant by removing the ORF of *alr3599*. | This study |
| *Δall4896* | A markerless deletion mutant by removing the ORF of *all4896*. | This study |
| *Δall5174* | A markerless deletion mutant by removing the ORF of *all5174*. | This study |
| *Δalr1230* | A markerless deletion mutant by removing the ORF of *alr1230*. | This study |
| *Δall0219* | A markerless deletion mutant by removing the ORF of *all0219*. | This study |
| *Δall1175* (*ΔcdgSH)* | A markerless deletion mutant by removing the ORF of *all1175* | This study |
| *Δall4225* | A markerless deletion mutant by removing the ORF of *all4225*. | This study |
| *Δall4897* | A markerless deletion mutant by removing the ORF of *all4897*. | This study |
| *Δalr2306* | A markerless deletion mutant by removing the ORF of *alr2306*. | This study |
| *Δalr3170* | A markerless deletion mutant by removing the ORF of *alr3170*. | This study |
| *Δalr3920* | A markerless deletion mutant by removing the ORF of *alr3920*. | This study |
| *ΔcdgSHΔcdgS* | A double markerless mutant by removing both *cdgSH* and *cdgS* ORF | This study |
| *CcdgSH* | *ΔcdgSH* mutant complemented by *cdgSH* | This study |
| *ΔcdgSH::yhjH* | The *yhjH* gene from *E. coli*, controlled by the *cdgSH* native promoter, expressed in the *ΔcdgSH* mutant*.* | This study |
| *ΔcdgSH::yhjH^AAA^* | Same as *ΔcdgSH::yhjH*, except that the active site of the corresponding PDE activity was mutated from ELL to AAA. | This study |
| *ΔcdgSH::ydeH* | The *ydeH* gene from *E. coli*, controlled by the *cdgSH* promoter, expressed in *ΔcdgSH* mutant. | This study |
| *ΔcdgS::ydeH* | The *ydeH* gene, controlled by the *cdgS* promoter, expressed in *ΔcdgS* mutant | This study |
| *ΔcdgS::ydeH^GGAAF^* | Same as *ΔcdgS::ydeH*, except that the active site of YdeH was changed from GGDEF to GGAAF | This study |
| OE_CT_-*cdgSH* | *cdgSH* overexpression strain, under the control of the CT promoter (inducible by copper and theophylline) | This study |
| OE_CT_-*cdgSH*^AAA^ | A strain overexpressing an inactive PDE domain (for c-di-GMP hydrolysis) of CdgSH under the control of the CT promoter | This study |
| OE_CT-_*cdgSH*^GGAAF^ | A strain overexpressing an inactive DGC domain (for c-di-GMP synthesis) of CdgSH under the control of the CT promoter | This study |
| OE_CT_-*cdgSH*^GGAAF-AAA^ | A strain overexpressing a form of CdgSH in which both the c-di-GMP synthesis and hydrolysis activities were abolished, under the control of the CT promoter | This study |
| OE_CT_-*cdgS* | *cdgS* overexpression strain, under the control of the CT promoter | This study |
| OE_CT_-*cdgS*^GGAAF^ | A strain overexpressing an inactive DGC domain (for c-di-GMP synthesis) of CdgS under the control of the CT promoter | This study |
| OE_CT_-*yhjH* | *yhjH* overexpression strain, under the control of the CT promoter | This study |
| OE_CT_-*yhjH*^AAA^ | A strain overexpressing an inactive PDE domain of YhjH, under the control of the CT promoter | This study |
| OE_CT_-*ydeH* | *yhjH* overexpression strain, under the control of the CT promoter | This study |
| OE_CT_-*ydeH*^GGAAF^ | A strain overexpressing an inactive DGC domain of YdeH, under the control of the CT promoter | This study |

**Table S2 Plasmids used in this study**

| **Plasmids** | **Description** | **Source** |
| --- | --- | --- |
| CRISPR-pCpf1b | Sp^r^ Sm^r^; vector carrying the CRISPR-pCpf1b genome editing system | (Niu *et al*., 2019) |
| pCpf1b-M*all1012*-F223 | Sp^r^ Sm^r^; for constructing *Δall1012* by CRISPR-pCpf1b genome editing system, origin from CRISPR-pCpf1b | This study |
| pCpf1b-M*all1219*-F172 | Sp^r^ Sm^r^; for constructing *Δall1219* by CRISPR-pCpf1b genome editing system, origin from CRISPR-pCpf1b | This study |
| pCpf1b-M*all2416*-F103 | Sp^r^ Sm^r^; for constructing *Δall2416* by CRISPR-pCpf1b genome editing system, origin from CRISPR-pCpf1b | This study |
| pCpf1b-M*cdgS*-F149 | Sp^r^ Sm^r^; for constructing *ΔcdgS* by CRISPR-pCpf1b genome editing system, origin from CRISPR-pCpf1b | This study |
| pCpf1b-M*alr3504*-F100 | Km^r^ Nm^r^; for constructing *Δalr3504* by CRISPR-pCpf1b genome editing system, origin from CRISPR-pCpf1b | This study |
| pCpf1b-M*alr3599*-F489 | Sp^r^ Sm^r^; for constructing *Δalr3599* by CRISPR-pCpf1b genome editing system, origin from CRISPR-pCpf1b | This study |
| pCpf1b-M*all4896*-F119 | Sp^r^ Sm^r^; for constructing *Δall4896* by CRISPR-pCpf1b genome editing system, origin from CRISPR-pCpf1b | This study |
| pCpf1b-M*all5174*-F147 | Sp^r^ Sm^r^; for constructing *Δall5174* by CRISPR-pCpf1b genome editing system, origin from CRISPR-pCpf1b | This study |
| pCpf1b-M*alr1230*-F543 | Sp^r^ Sm^r^; for constructing *Δalr1230* by CRISPR-pCpf1b genome editing system, origin from CRISPR-pCpf1b | This study |
| pCpf1b-M*all0219*-F270 | Sp^r^ Sm^r^; for constructing *Δall0219* by CRISPR-pCpf1b genome editing system, origin from CRISPR-pCpf1b | This study |
| pCpf1b-M*cdgSH*-R2389 | Sp^r^ Sm^r^; for constructing *ΔcdgSH* by CRISPR-pCpf1b genome editing system, origin from CRISPR-pCpf1b | This study |
| pCpf1b-M*all4225*-F192 | Sp^r^ Sm^r^; for constructing *Δall4225* by CRISPR-pCpf1b genome editing system, origin from CRISPR-pCpf1b | This study |
| pCpf1b-M*all4897*-F204 | Sp^r^ Sm^r^; for constructing *Δall4897* by CRISPR-pCpf1b genome editing system, origin from CRISPR-pCpf1b | This study |
| pCpf1b-M*alr2306*-F580 | Sp^r^ Sm^r^; for constructing *Δalr2306* by CRISPR-pCpf1b genome editing system, origin from CRISPR-pCpf1b | This study |
| pCpf1b-M*alr3170*-F127 | Sp^r^ Sm^r^; for constructing *Δalr3170* by CRISPR-pCpf1b genome editing system, origin from CRISPR-pCpf1b | This study |
| pCpf1b-M*alr3920*-F86 | Sp^r^ Sm^r^; for constructing *Δalr3920* by CRISPR-pCpf1b genome editing system, origin from CRISPR-pCpf1b | This study |
| pRL25 | Km^r^ Nm^r^; A cosmid vector, containing replicons functional in *Escherichia coli* and in *Anabaena* | (Wolk *et al*., 1988) |
| pRL-*cdgSH* | Km^r^ Nm^r^; for constructing *cdgSH* complementary strain | This study |
| pRL-P*cdgSH-yhjH* | Km^r^ Nm^r^; for *yhjH* expression that controlled by the native promoter of *cdgSH* | This study |
| pRL-P*cdgSH-yhjH^AAA^* | Km^r^ Nm^r^; for expressing inactivated PDE *yhjH* that controlled by *cdgSH* native promoter | This study |
| pRL-P*cdgSH-ydeH* | Km^r^ Nm^r^; for expressing *ydeH* that controlled by *cdgSH* native promoter | This study |
| pRL-P*cdgSH-ydeH^GGAAF^* | Km^r^ Nm^r^; for expressing DGC inactivated *ydeH* that controlled by *cdgSH* native promoter | This study |
| PCT | Km^r^ Nm^r^; expression plasmid under the control of the CT promoter | (Xing *et al*., 2020) |
| PCT-*cdgSH* | Km^r^ Nm^r^; for constructing *cdgSH* overexpression strain | This study |
| PCT-*cdgSH^AAA^* | Km^r^ Nm^r^; for constructing PDE inactivated *cdgSH* overexpression strain | This study |
| PCT-*cdgSH^GGAAF^* | Km^r^ Nm^r^; for constructing DGC inactivated *cdgSH* overexpression strain | This study |
| PCT-*cdgSH^GGAAF-AAA^* | Km^r^ Nm^r^; for constructing both DGC and PDE inactivated *cdgSH* overexpression strain | This study |
| pRL-P*cdgS-ydeH* | Km^r^ Nm^r^; for expressing *ydeH* that controlled by *cdgS* native promoter | This study |
| pRL-P*cdgS-ydeH^GGAAF^* | Km^r^ Nm^r^; for expressing inactivated DGC *ydeH* that controlled by *cdgS* native promoter | This study |
| PCT-*cdgS* | Km^r^ Nm^r^; for constructing *cdgS* overexpression strain | This study |
| PCT-*cdgS^GGAAF^* | Km^r^ Nm^r^; for constructing inactivated DGC *cdgS* overexpression strain | This study |
| PCT-*yhjH* | Km^r^ Nm^r^; for constructing *yhjH* overexpression strain | This study |
| PCT-*yhjH^AAA^* | Km^r^ Nm^r^; for constructing inactivated PDE *yhjH* overexpression strain | This study |
| PCT-*ydeH* | Km^r^ Nm^r^; for constructing *ydeH* overexpression strain | This study |
| PCT-*ydeH^GGAAF^* | Km^r^ Nm^r^; for constructing inactivated DGC *ydeH* overexpression strain | This study |
| pHTS-*cdgSH* | Km^r^ ; protein expression plasmid containing the C-terminal Strep-tagged *cdgSH* ORF; | This study |
| pHTS-*cdgSH^AAA^* | Km^r^ ; protein expression plasmid containing the C-terminal Strep-tagged inactivated PDE *cdgSH* ORF; | This study |
| pHTS-*cdgSH^GGAAF^* | Km^r^ ; protein expression plasmid containing the C-terminal Strep-tagged inactivated DGC *cdgSH* ORF; | This study |
| pHTS-*cdgS* | Km^r^ ; protein expression plasmid containing the C-terminal Strep-tagged *cdgS* ORF; | This study |
| pHTS-*cdgS^GGAAF^* | Km^r^ ; protein expression plasmid containing the C-terminal Strep-tagged inactivated DGC *cdgS* ORF; | This study |

**Table S3. Primers used in this study (sequences in lower case correspond to the overlapping homologous parts in PCR fragments for ligation during cloning, and the sequence in capital corresponds to those used for DNA amplification by PCR).**

| Name | Sequence (**5’-3’**) |
| --- | --- |
| Pall1012F1050m | gcagaaattcgatatctagatct ATAACCCGGATGGGGTGGCTA |
| Pall1012R1m | GTTAAAATTACCTGTTTGCTAAGGTTCA |
| Pall1012F1268 | tgaaccttagcaaacaggtaattttaacATGGTGTATTTAATAAAGCCGCAAA |
| Pall1012R2238 | cgcaacgttgttgccattgcTGGCTCCAGATGATGAGTGTT |
| cr_ all1012F223F | agatGCATACCAGTATTATATGTAGC |
| cr_all1012F223R | agacGCTACATATAATACTGGTATGC |
| Pall1219F1034m | gcagaaattcgatatctagatct ATCGTGGGCATGGTAACGCT |
| Pall1219R1m | TCCCAATACCTAAGTGGGATAATTGTC |
| Pall1219F2186 | gacaattatcccacttaggtattgggaTGGCGGATAGGGTAAATACTGAGT |
| Pall1219R3199 | cgcaacgttgttgccattgc ATTACCGCAGCTACGGAACT |
| cr_ all1219F172F | agatGCCTCCGACCAAACGAACAGCC |
| cr_all1219F172R | agacGGCTGTTCGTTTGGTCGGAGGC |
| Pall2416F1123m | gcagaaattcgatatctagatct TGTAGGTAGACTTACCTCCGCA |
| Pall2416R1m | GGCTGTATTTTTTGCCTTTTTTCTAGC |
| Pall2416F994 | gctagaaaaaaggcaaaaaatacagccTGATAGTGTTCTGCCTACCGCT |
| Pall2416R1973 | cgcaacgttgttgccattgcATCCGGAGAATAAGAGGAAATCTGAC |
| cr_ all2416F103F | agatATCAGGCCGCATCGCAGATCCA |
| cr_all2416F103R | agacTGGATCTGCGATGCGGCCTGAT |
| PcdgSF999m | gcagaaattcgatatctagatctACACCTACCCTTGGTGATTT |
| PcdgSR1ma | GATGTCTCCTTGTTCACTTCT |
| PcdgSF994a | agaagtgaacaaggagacatcGCAAGAAGTTAGAAGCTAAGGT |
| PcdgSR1992 | cgcaacgttgttgccattgc AAGGGCTAAAGCTTTTGGGA |
| cr_cdgSF149F | agatAAGAACTGCAACCTGATATCGT |
| cr_cdgSF149R | agacACGATATCAGGTTGCAGTTCTT |
| Palr3504F1037m | gcagaaattcgatatctagatctTCAACGGTACTCAGCACTGATT |
| Palr3504R1m | AACACGACGGCTGTTGTTATC |
| Palr3504F891 | gataacaacagccgtcgtgttGAGTGAAGAATGGAAGGTTGTGAA |
| Palr3504R1896 | cgcaacgttgttgccattgcAGCCGCCAATACCCCTGA |
| cr_ alr3504F100F | agatCGGTTGGTCTCATAGGGTTAAT |
| cr_alr3504F100R | agacATTAACCCTATGAGACCAACCG |
| Palr3599F232m | gcagaaattcgatatctagatctTCCAACCTAGTATTACTCCAGATTCT |
| Palr3599R1m | GAGATGCGTTCCGTAGAAAATCAG |
| Palr3599F1033 | ctgattttctacggaacgcatctcTAGTTTTCTTTTCCCCTTGCGCCCTA |
| Palr3599R2026 | cgcaacgttgttgccattgcAGCCGGTAGTAACATCATCACC |
| cr_ alr3599F489F | agatGAAGCTGCGAACATGGCCTTAC |
| cr_alr3599F489R | agacGTAAGGCCATGTTCGCAGCTTC |
| Pall4896F949m | gcagaaattcgatatctagatctGCAGCAGATGAATATTCAGTTACAC |
| Pall4896R1m | TAGTCACTTAAGATAGCAATACTATTAGAG |
| Pall4896F1753 | taatagtattgctatcttaagtgactaATCTCTTAAGTTAGCAACTTATATGCAGA |
| Pall4896R2699 | cgcaacgttgttgccattgcTGTAACGGGCAATATCCACAATG |
| cr_ all4896F119F | agatAAGGAGATGCCCGCGAGCTTCA |
| cr_ all4896F119R | agacTGAAGCTCGCGGGCATCTCCTT |
| Pall5174F1010m | gcagaaattcgatatctagatct CCTGGTTCAAGAGAAGTCCTGC |
| Pall5174R1m | GGCGGTAAATCACCGCAAT |
| Pall5174F1803 | attgcggtgatttaccgcc TGAATCAGGTAAGGAACAGGAGAA |
| Pall5174R2760 | cgcaacgttgttgccattgc CAATACGCAGGCGATCGTCT |
| cr_ all5174F147F | agatGATTTGATCTTGCCTAAGCTGG |
| cr_all5174F147R | agacCCAGCTTAGGCAAGATCAAATC |
| Palr1230F1000m | gcagaaattcgatatctagatctATAACTGGCTATCCAGCCCAAG |
| Palr1230R8 | TTAATCATTTTTGACCTCTTCGTTTATTGA |
| Palr1230F1216 | tcaataaacgaagaggtcaaaaatgattaaATAGCCAAATGACCACTACCGT |
| Palr1230F2209 | cgcaacgttgttgccattgcTAACTAGGCTGCCTGGAGAG |
| cr-alr1230F543F | agatTTACGTTGGAACAGTCCTGAAT |
| cr-alr1230F543R | agacATTCAGGACTGTTCCAACGTAA |
| Pall0219F963m | gcagaaattcgatatctagatctAGTGATTGTACTAGATCGCATCCAG |
| Pall0219R1m | GCAGATGACGCTCCGTGTTG |
| Pall0219F1756 | caacacggagcgtcatctgcTAGGCGTGATTTTTAGGCGAT |
| Pall0219R2723 | cgcaacgttgttgccattgcTTTCCAGATTACGCAGCTATCC |
| cr_ all0219F270F | agatGGCGCAACTGTACTAGTAGAGT |
| cr_all0219F270R | agacACTCTACTAGTACAGTTGCGCC |
| PcdgSHF943m | gcagaaattcgatatctagatc CTGCAAGCCAGAAACGAATC |
| PcdgSHR17a | CACTCGTTGCCTGGCATT |
| PcdgSHF2647g | aatgccaggcaacgagtg CGAGTACAGGTAATAGCTCATTGG |
| PcdgSHR3638 | cgcaacgttgttgccattgc GTCTTCTCTATGCAGCTGCTTG |
| cr_cdgSHF2389F | agatGATTGTGGAGTGGTAAAAGCTG |
| cr_cdgSHR2389R | agacCAGCTTTTACCACTCCACAATC |
| Pall4225F1055m | gcagaaattcgatatctagatct AGTCGTCAATCTTCCACTTTGC |
| Pall4225R1m | AGATCCTAATGTGGGGTTTGAAAG |
| Pall4225F1673 | ctttcaaaccccacattaggatct ACCAAAAGAGGCGATAGCAGTC |
| Pall4225R2694 | cgcaacgttgttgccattgc CGTCTCTGGATTGCTACTCCCT |
| cr_ all4225F192F | agatTTCGCTACGTTAATTGGTACGG |
| cr_all4225F192R | agacCCGTACCAATTAACGTAGCGAA |
| Pall4897F1027m | gcagaaattcgatatctagatctGCAAACTCAGCAAATCAATGTTGT |
| Pall4897R1m | AGACCAGTTCATTTTTCGTGCCTA |
| Pall4897F2467 | taggcacgaaaaatgaactggtct TCCCTGTTAGCTTCGTCTGA |
| Pall4897R3464 | cgcaacgttgttgccattgcGCTCCAAATGGGCCTTGATCTA |
| cr_ all4897F204F | agatTCTCCTCACGGGTAAACCCGCA |
| cr_all4897F204R | agacTGCGGGTTTACCCGTGAGGAGA |
| Palr2306F996m | gcagaaattcgatatctagatct AGCGTCAAAATACATTGCCGGAA |
| Palr2306R35 | TCGCTTTTATATGGGTCTAACTGT |
| Palr2306F1820 | acagttagacccatataaaagcgaAGCTACCCAATGGTAGTTTAGGTTGA |
| Palr2306R2761 | cgcaacgttgttgccattgc AGCGCCAGTACAATTTCCCGAA |
| cr_ alr2306F580F | agatTCAGGAGATTAGGAAAAGCTTT |
| cr_alr2306F580R | agacAAAGCTTTTCCTAATCTCCTGA |
| Palr3170F1029m | gcagaaattcgatatctagatctACACCCTGTACATCAGTTATTTGG |
| Palr3170R1m | TGTCAGATTTTTGTAAATTGCTGAGG |
| Palr3170F3064 | cctcagcaatttacaaaaatctgacaTAGAACACAAAATCTTGGCTTGTCC |
| Palr3170R4069 | cgcaacgttgttgccattgcCATCAATGCGGGATGATTTCTATCA |
| cr_ alr3170F127F | agatAGTCTTATGAAAACTCCATATT |
| cr_alr3170F127R | agacAATATGGAGTTTTCATAAGACT |
| Palr3920F980m | gcagaaattcgatatctagatct ACACGCGAGTGGCTTGTTAG |
| Palr3920R3 | CACAGAAGTACCCCAATCGTTTA |
| Palr3920F1236 | taaacgattggggtacttctgtg GCTGAGTAGTGAATGCTGAGTAAG |
| Palr3920R2223 | cgcaacgttgttgccattgc CTCCCGGATTGGATCACAGTA |
| cr_ alr3920F86F | agatAAATCGCGTTTCCGGAGTACAA |
| cr_ alr3920F86R | agacTTGTACTCCGGAAACGCGATTT |
| Pall1012F1081m | ACTATGCGGTGATGTCCAGAG |
| Pall1012R2298 | GGAAGAAGAAGTCTCGCAAAACC |
| Pall1219F1083m | TCTTGGGCGCTCTTGGGAAG |
| Pall1219R3320 | GAAAGTTGCACACGGCGTTA |
| Pall2416F1278m | TCCCATTCCCCAGTTCCCA |
| Pall2416R2234 | CTGGTGTAGCGATATCAACGATA |
| PcdgSF1048m | TGGACGGTCTGACACTGGCA |
| PcdgSR2036 | GTTGGGCAGAAAGAAGCGGA |
| Palr3504F1127m | AGTTGTTTCCTTGAGGCTTGATG |
| Palr3504R1938 | AACCCCGTACTGTCCAGATG |
| Palr3599F478m | AGTGAGAGCAGTCCTGGGT |
| Palr3599R2349 | CCATCGACACCTGTCAGTCTAT |
| Pall4896F989m | TGGAGAACCATGAATCAGCCA |
| Pall4896R2793 | GGATGTAAATTGACCCGTGACA |
| Pall5174F1074m | AACAATAATGGGCTGTGGGAATA |
| Pall5174R2830 | ATTGGGATGGCGAAAACGAT |
| Palr1230F1110m | TCAGCCAACAGCGACTACA |
| Palr1230R2285 | TGTGCTGCTGTAATTCCTCG |
| Pall0219F1010m | GGTAAGACAGATCGCTCCGT |
| Pall0219R2790 | CTGCACCCAAAGTGGAAGGT |
| PcdgSHF1013m | GTAGCCAGTCTGATGACACT |
| PcdgSHR3674 | CAGAGGGTTAACCGGACTTG |
| Pall4225F1101m | TCCACCTGTCGCCCTGTCA |
| Pall4225R2767 | TCCTACAGGCCCCGCAGAT |
| Pall4897F1066m | TCACTGCCCCGTGGTACAT |
| Pall4897R3600 | TACCAGCCGCCCTTGTTCTG |
| Palr2306F1076m | CTGCCAGCGTTGTGTAGTTC |
| Palr2306R2850 | AGGCGCTGTCTATTCCCTGAT |
| Palr3170F1092m | AGGTCGCTGGCGAATACTG |
| Palr3170R4122 | ACCCAGGGTATACAGGGAACA |
| Palr3920F1030m | TGTCCGCAATTATGGAGTGTG |
| Palr3920R2281 | GACTGTGACATTTGCACCCA |
| PyhjHF3 | ATAAGGCAGGTTATCCAGCGAA |
| PyhjHR768 | TTATAGCGCCAGAACCGCCGTA |
| P25T-yhjHF | tacggcggttctggcgctataa GCGGCCGCAAGCCCAAGC |
| P25T-yhjHR | ttcgctggataacctgccttat TTTCTCCCACTCGTTGCCTG |
| P25T-yhjHFa | cgaacaaaatgtgattaaccgagtttaa GCGGCCGCAAGCCCAAGC |
| P25T-yhjHRa | catcaatttccgttgtcttcttgat TTTCTCCCACTCGTTGCCTG |
| PydeHF627 | ggcgcagcATTTATCATTATTGTCAAAGCGGCT |
| PydeHR622 | aaatgctgCGCCCCCGTAGCGATAAA |
| PydeHF3 | ATCAAGAAGACAACGGAAATTGATGC |
| PydeHR891 | TTAAACTCGGTTAATCACATTTTGTTCG |
| PyhjHF150 | gcggcagc AACGGTGGTCACGCATCCCTTG |
| PyhjHR141 | gctgccgc CACGGCCATTAACCGCCCGCAT |
| PcdgSHF600mb | caggttaggagaacgccatgAATTGAACTCAGCATGACACAG |
| PcdgSHR2649 | ataacaaaacgcttgggcttTCGATATTACAGAGTAGCGTGT |
| PcdgSHF4 | aaggaggtaacaacaagatg CCAGGCAACGAGTGGGAG |
| PcdgSHR2649 | ataacaaaacgcttgggctt TCGATATTACAGAGTAGCGTGT |
| PcdgSHF4c | gtgccgcgcggcagcCCAGGCAACGAGTGGGAG |
| PcdgSHR2640c | accaccaccagaaccCAGAGTAGCGTGTTTTTTTTCAC |
| PcdgSHF1974 | gcagcagcGTTACGTTGGCAACATCCAGAG |
| PcdgSHR1966 | cgctgctgCCATACCAGTAATTTGTCCGGTGA |
| PcdgSHF1588 | ggtgctgcATTTACAATTCTTCTGCCCCACAT |
| PcdgSHR1593 | aaatgcagCACCTCCCCAACGAGAAAC |
| PcdgSF4c | ggtaacaacaagatg AAAAATACAGTTCCAGAGAGCCA |
| PcdgSR990c | accaccagaaccccc AGCTAGTCGATGGTGAGC |
| PcdgSF4b | gtgccgcgcggcagc AAAAATACAGTTCCAGAGAGCCA |
| PcdgSR990b | accaccaccagaacc AGCTAGTCGATGGTGAGC |
| PcdgSF685 | ggcgcagcATTTGCTGTGATCCTACCCA |
| PcdgSR689 | aaatgctgCGCCACCATAACGAGCGA |
| PV_6 | GGTTCTGGTGGTGGTAGCA |
| PV_1 | GCTGCCGCGCGGCACCAG |
| PV_20 | GGGGGTTCTGGTGGTGGTAGCACT |
| PV_19 | CATCTTGTTGTTACCTCCTTAGCA |
| Pallrs04F334 | CCAGTTCCGCTATCAGAGAG |
| Pallrs04R456 | GAGGAGAGAGTTGGTGGTAAG |
| PcdgSHF2342 | CTGGTCATTCTTCCTTATC |
| PcdgSHR2455 | CCTTAACTATATGTGCTACTT |
| PcdgS4F226 | CAGTTATTAGAAGACGGTAA |
| PcdgSR402 | TTGTTGGAGATGGAATTG |

**
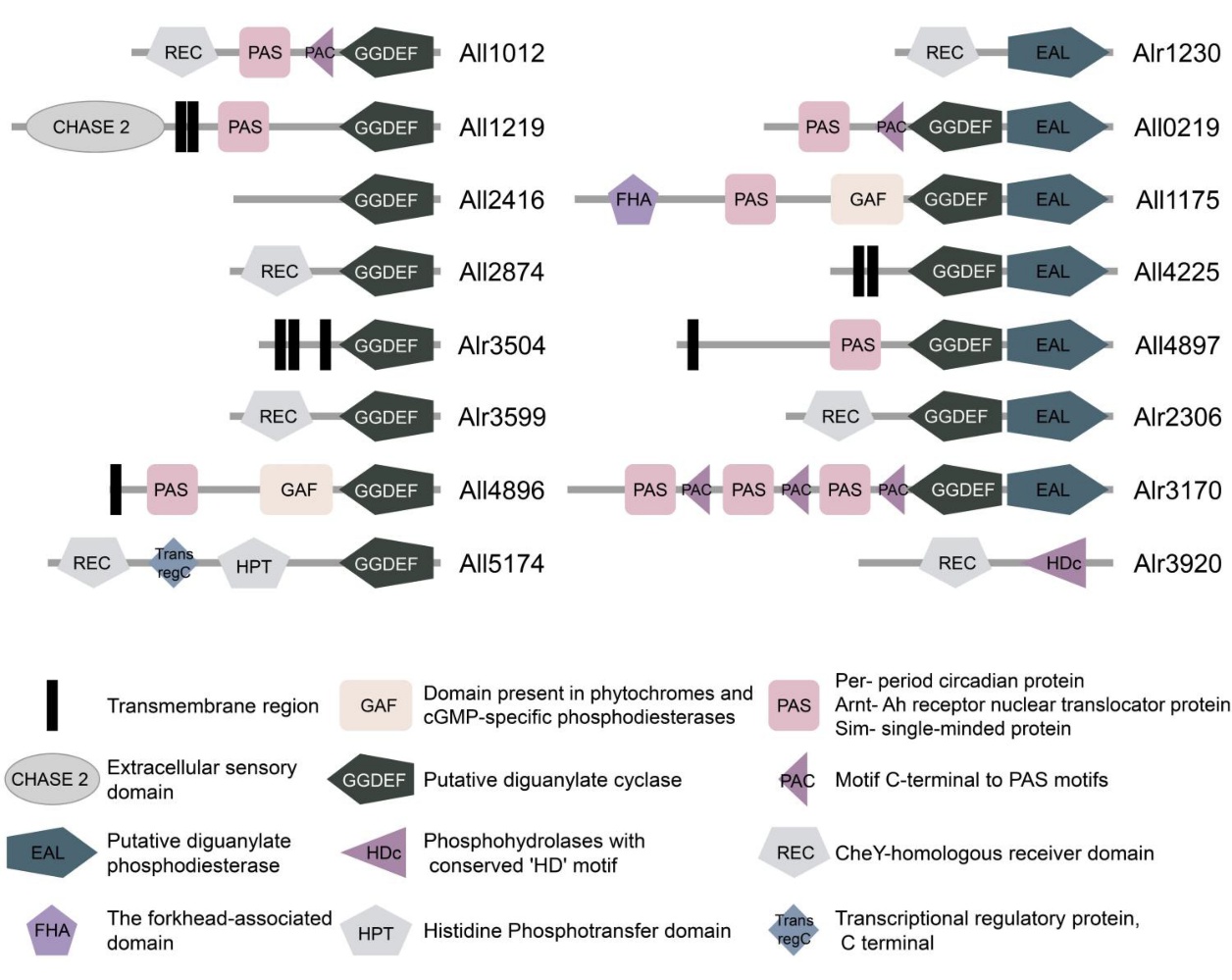
**

**Fig S1** Putative domain architectures of 16 proteins related to c-di-GMP metabolism in *Anabaena*. The domain symbols were deduced by SMART (smart.embl-heidelberg.de).


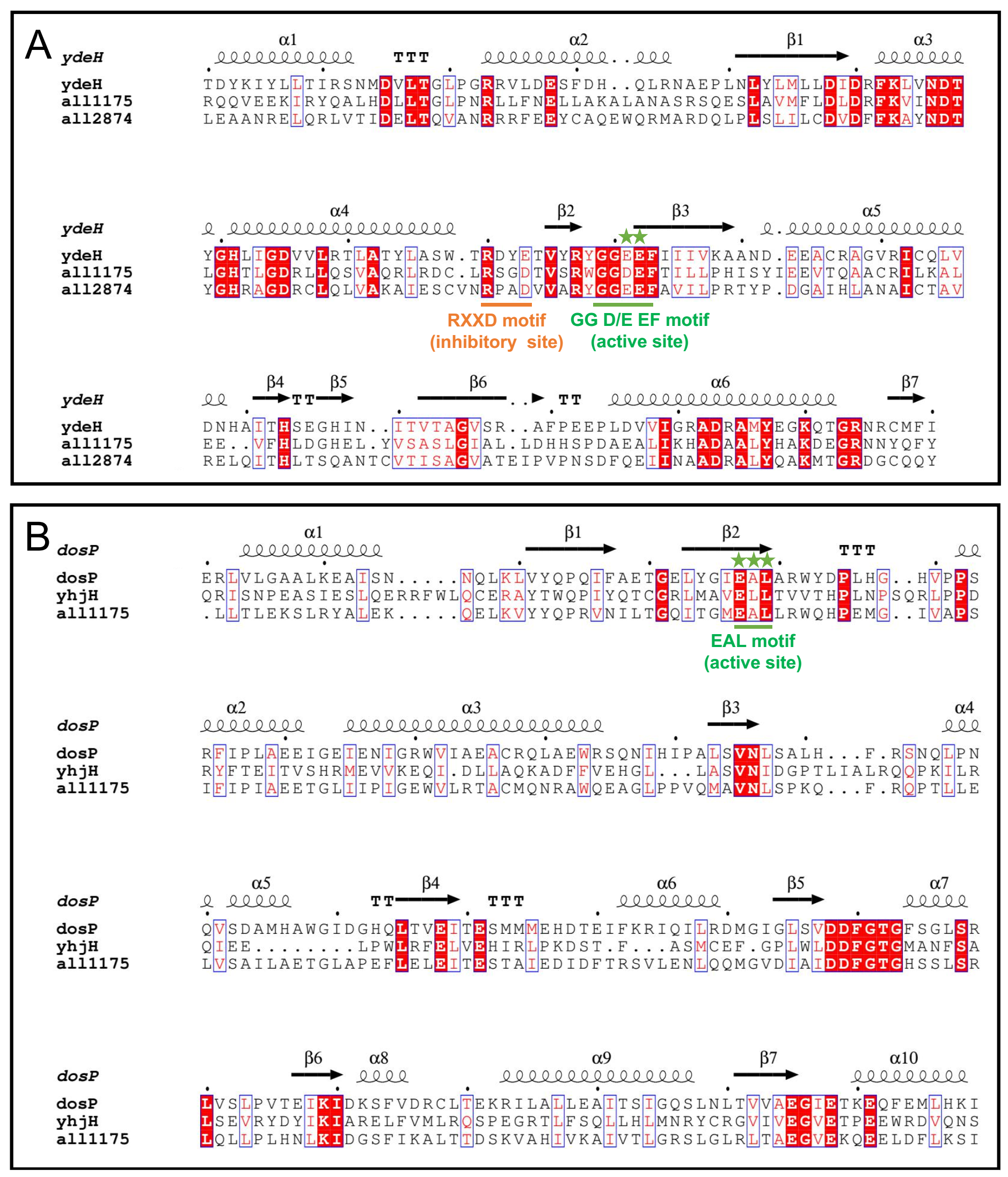


**Fig S2 Sequence alignment of GG (D/E) EF and EAL domains from different proteins used in this study.** The domains were analyzed by SMART (smart.embl-heidelberg.de). The sequences were aligned using CLUSTAL OMEGA and visualized with ESPript 3.X. Residues with identical residues are shown as white characters on a red background, whereas those with high similarity are shown as red characters and are framed. (A) Sequences alignment of GG (D/E) EF domains for the following proteins: CdgSH (All1175) from *Anabaena* (this study), CdgS (All2874) from *Anabaena* (this study), YdeH from *E. coli* (this study). The secondary-structure elements depicted above the alignment was generated by the crystal structure of YdeH EAL domain (PDB entry 4H54) by ESPript 3.X. The GG (D/E) EF motif was indicated by a green underline and the point mutation sites of the DGC inactive variant in this study were indicated by green asterisks. The inhibitory sites (RXXD motif) were indicated by an orange underline. (B) Sequences alignment of EAL domains for the following proteins: CdgSH from *Anabaena* (this study), YhjH from *E. coli* (this study) and DosP from E. coli (PDB entry 4HU3). The secondary-structure elements depicted above the alignment was generated by the crystal structure DosP EAL domain by ESPript 3.X. The EAL motif was indicated by a green underline and the point mutation sites of the PDE inactive variant in this study were indicated by green asterisks.


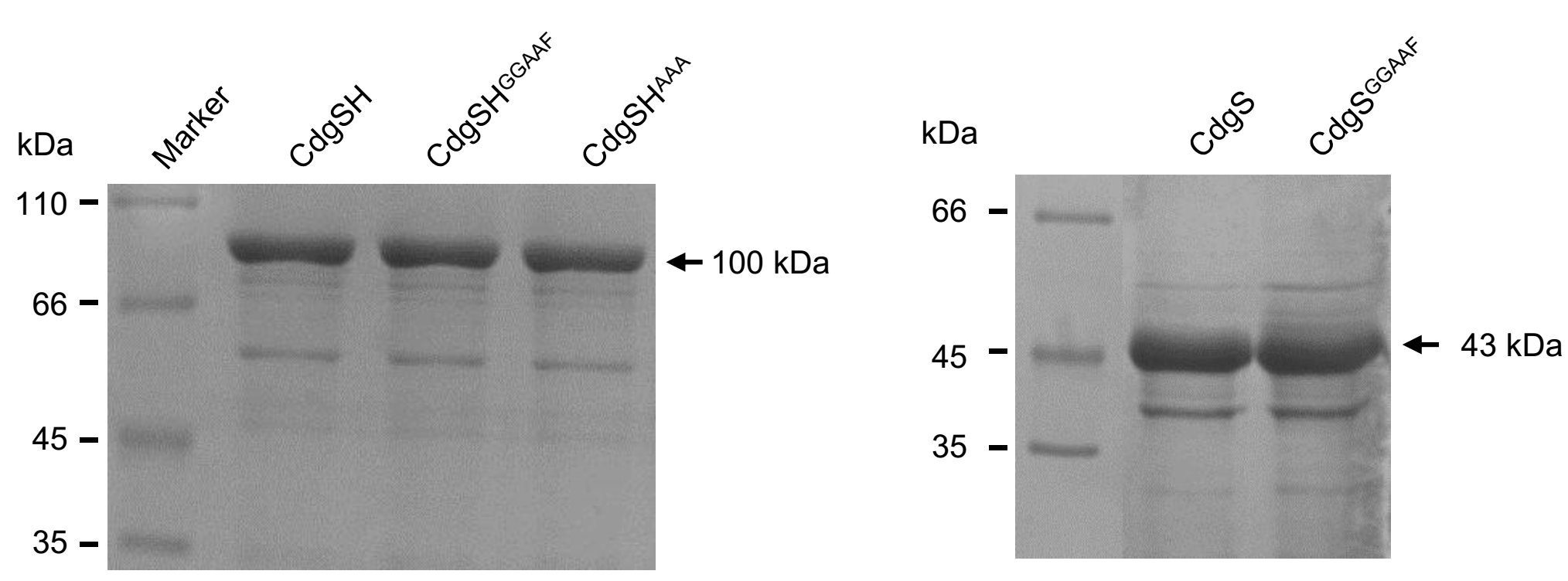


**Fig S3 SDS-PAGE of purified proteins used for DGC and PDE activity assay and for the steady-state kinetic parameters quantification.** The band for indicating proteins are point out by arrow.


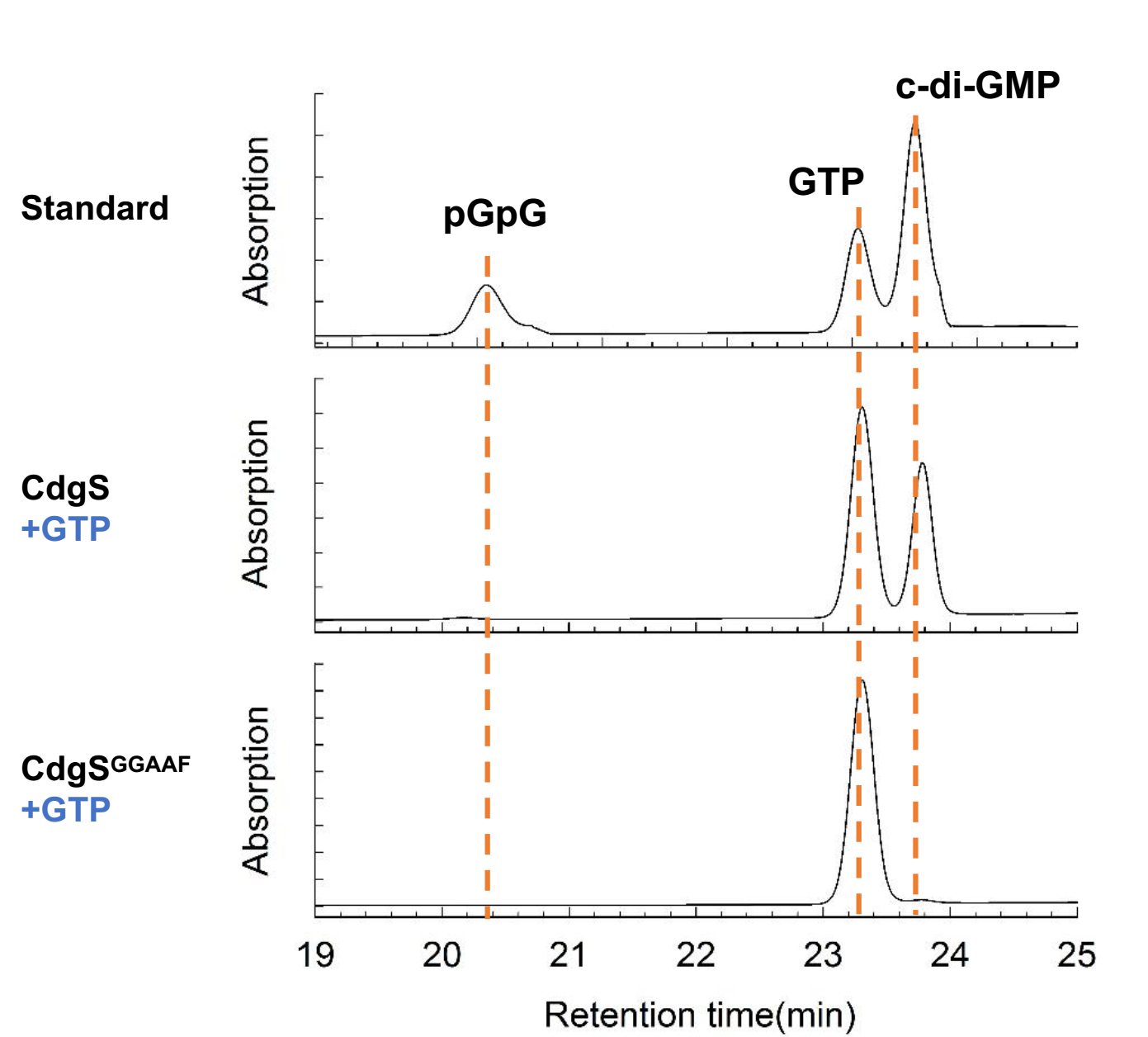


**Fig S4 *Anabaena* Protein CdgS has DGC activities.** The DGC (GTP→ c-di-GMP) activities of CdgS and its DGC inactive variant CdgS^GGAAF^ were evaluated by the reaction products. All reaction products are assessed by the retention time of HPLC and corresponded to standards. The top panel show the retention time of the standard nucleotides, including pGpG, c-di-GMP, GTP. The retention time of each nucleotide was first determined by HPLC separately (data not shown here).The protein used for DGC activity assay was indicated at the left of the chromatograms and the substrate for each reaction was indicated under the name of Protein in blue. CdgS can convert substrate GTP to c-di-GMP. The CdgS^GGAAF^, which inactive the DGC activity of CdgS, cannot synthesis c-di-GMP.


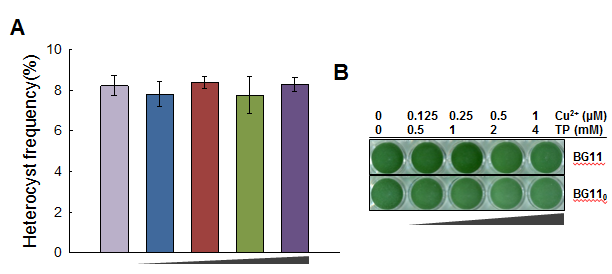


**FIG S5 Heterocyst frequcy and gowth of WT Anabaena with inducers.** (A) Heterocyst frequency of WT strains at 24 h after nitrogen stepdown with five concentrations of inducers. The black triangle indicated the increasing levels of the inducers added to the culture media (from left to right column): 0 μM copper and 0 mM theophylline, 0.125 μM copper and 0.5 mM theophylline, 0.25 μM copper and 1 mM theophylline, 0.5 μM copper and 2 mM theophylline, and 1 μM copper and 4 mM theophylline. (B) The growth of WT in BG11 and BG11_0_ medium with five concentrations of inducers tested in 24-well plates. All cultures were started with a similar OD at 0.3 diluted from a pre-culture and imaged after 4 days of incubation.

**
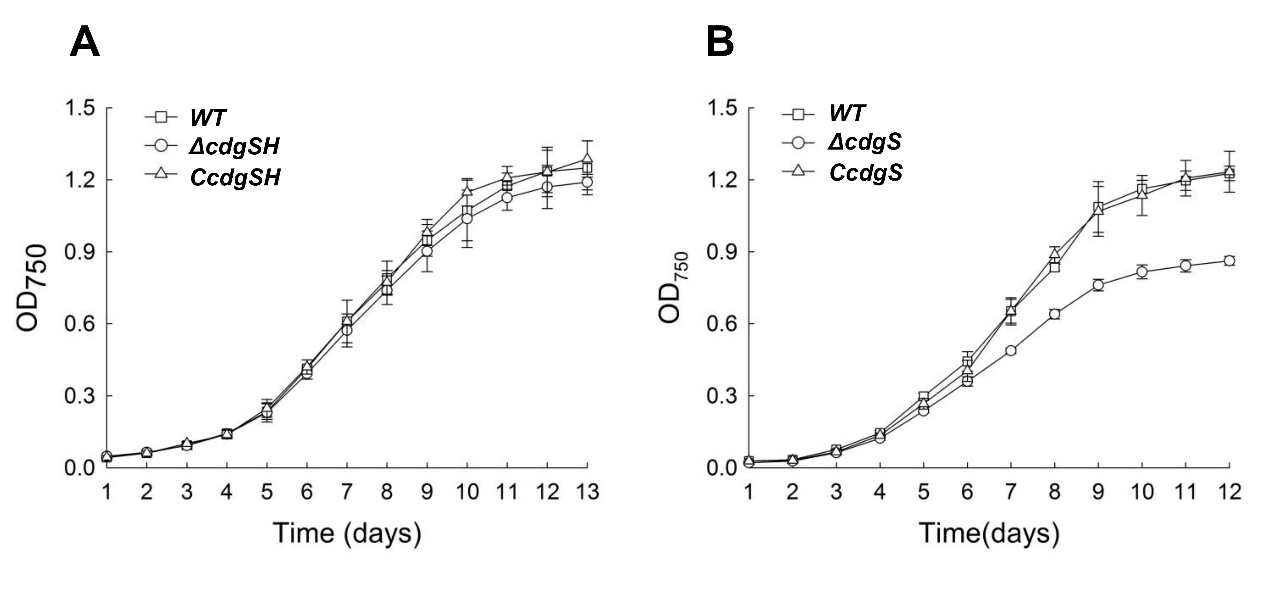
**

**FIG S6 Growth of WT *Anabaena*, deletion strain, and complemented strain in BG11_0_ medium.** (A). Growth of indicated strains, the white square is WT, the white circle is *cdgSH-deletion strain (ΔcdgSH),* the white triangle is complemented strain (C*cdgSH*). (B). Growth of indicated strains, the white square is WT, the white circle is *cdgS-deletion strain (ΔcdgS),* the white triangle is complemented strain (C*cdgS*).

**References**

ElhaI, J., Vepritskiy, A., MuroPastor, A.M., Flores, E., and Wolk, C.P. (1997). Reduction of conjugal transfer efficiency by three restriction activities of *Anabaena* sp. strain PCC 7120. *J. Bacteriol.* 179(6)**,** 1998-2005. doi: 10.1128/jb.179.6.1998-2005.1997.

Niu, T.C., Lin, G.M., Xie, L.R., Wang, Z.Q., Xing, W.Y., Zhang, J.Y., et al. (2019). Expanding the potential of CRISPR-cpf1-based genome editing technology in the cyanobacterium *Anabaena* PCC 7120. *ACS. Synth. Biol*. 8(1)**,** 170-180. doi: 10.1021/acssynbio.8b00437.

Wolk, C.P., Cai, Y.P., Cardemil, L., Flores, E., Hohn, B., Murry, M. et al. (1988) Isolation and complementation of mutants of *Anabaena* sp strain PCC 7120 unable to grow aerobically on dinitrogen. *J Bacteriol* **170**: 1239-1244. doi:10.1128/jb.170.3.1239-1244.1988

Xing, W.Y., Xie, L.R., Zeng, X.L., Yang, Y.L., and Zhang, C.C. (2020). Functional dissection of genes encoding dna polymerases based on conditional mutants in the heterocyst-forming cyanobacterium *Anabaena* PCC 7120. *Front. Microbiol.* 11. doi: 10.3389/fmicb.2020.01108.
